# Exploring Medicines Optimisation and Safety in the Community Following Mental Health Hospital Discharge: A Qualitative Interview Study

**DOI:** 10.1111/hex.70535

**Published:** 2025-12-25

**Authors:** Mark Jeffries, Fiona Naylor, Natasha Tyler, Catherine Robinson, Maria Panagioti, Richard N. Keers

**Affiliations:** ^1^ Division of Pharmacy and Optometry, School of Health Sciences, Faculty of Biology Medicine and Health University of Manchester Manchester UK; ^2^ NIHR School for Primary Care Research, Division of Population Health, Health Services Research and Primary Care School of Health Sciences University of Manchester Manchester UK; ^3^ NIHR Greater Manchester Patient Safety Research Collaboration University of Manchester Manchester UK; ^4^ Division of Nursing, Midwifery & Social Work, School of Health Sciences, Faculty of Biology, Medicine and Health University of Manchester Manchester UK; ^5^ Optimising Outcomes with Medicines (OptiMed) Research Unit Pennine Care NHS Foundation Trust Manchester UK

**Keywords:** co‐ordination of care, discharge, medication safety, medicines optimisation, mental health, primary care, transition of care

## Abstract

**Background:**

Medication safety issues may be common following mental health hospital discharge. However, there is little research exploring their aetiology and influencing factors for patients with mental illness and their carers in the community. This study aimed to explore medicine taking, continuity and care following mental health hospital discharge from the perspectives of people with lived experience, their carers and community‐based health professionals.

**Method:**

Participant recruitment was via social media and the professional networks of the research team. Semi‐structured online interviews were conducted with people with mental illness, carers and health professionals (including pharmacists, doctors and nurses in hospital, primary care and community settings) involved in medication use and safety after hospital discharge. Questions focused on medication‐related activities, knowledge transfer practice, support needs and key challenges and facilitators of medication safety in the community following discharge. Template analysis involved independent reading, development of a coding framework and coding across the research team.

**Results:**

Analysis of 34 interviews conducted with 17 healthcare professionals, 10 people with lived experience and 7 carers suggested a fragmented and disrupted network of care provision following discharge. This could include diversion of responsibility, lack of collaboration and continuity of care. People with lived experience and carers reported poor provision of information about medications, variable shared decision‐making, and feelings of disempowerment and not being listened to.

**Conclusion:**

This study has revealed the system‐level challenges associated with maintaining safety with medicines following mental health hospital discharge, particularly around the continuity of care and disempowerment of patients. We suggest that clearer pathways and the organisation of care services based around collaborative working would benefit medicines management post‐discharge. Further attention is needed to develop care provision that is holistic and person‐centred.

**Patient and Public Contribution:**

The research team included a person with lived experience. They took part in all aspects of the study, including data analysis, and are co‐authors. In addition, our study advisory group included a person with lived experience and a carer.

## 1 Introduction

Mental illnesses are a leading cause of morbidity in England [1, 2] and were associated with costs in excess of £100 billion in 2019 [2]. If symptoms are severe or pose a serious risk, patients may be admitted to hospital voluntarily, or under the Mental Health Act in England and Wales. In recent years, there has been increased use of the Mental Health Act to detain people in hospital [3, 4]. Treatment changes, including changes to medications, during hospitalisation, and being discharged without appropriate support may make discharge challenging [5, 6], and patients often find transitions of care disruptive and emotionally difficult [6]. Patients have been said to not have their voices heard during transitions, leading to a sense of being ‘lost’ in the system [6].

The time following discharge from acute mental health inpatient care is considered particularly high risk. Re‐admission rates following discharge are high [7], and recent studies have highlighted an increased risk of mortality, self‐harm and crime following mental health hospital discharge relative to the general population [8, 9, 10]. Patients report feeling unheard during discharge, despite policies promoting inclusion and shared decision‐making in mental healthcare [5]. Patient safety incidents are also common during care transitions and may be related to issues concerning continuity of care and information sharing, delayed discharge and medication management [11, 12, 13].

As medicines are the most common treatment intervention for those with mental illness, related safety issues may be common at and following discharge, particularly since medications often change from those taken pre‐admission [14, 15]. More than one in twenty prescribed medications from mental health hospitals may contain errors, with discontinued medicines omitted from two‐thirds of communications to primary care [16]. One study reported that 43% of medicines issued in primary care following discharge were different from discharge summaries, of which almost 40% were erroneous [17]. Unsafe discharges can burden primary care with correcting medication issues and may contribute to mental health hospital readmissions [18].

The World Health Organization's Third Global Patient Safety Challenge ‘Medication without Harm’ made improving care transitions one of its three priorities for action [19]. Elsewhere, stakeholders have raised continuity of care as an important area for safety improvement [20], with one survey highlighting medication management at discharge as a key safety priority for healthcare professionals, with patients conceptualising post‐discharge safety primarily in terms of information provision and social support that accompanies medication management [21].

Whilst an established evidence base exists in non‐mental health acute care for understanding and improving medication safety after discharge [22, 23, 24, 25], research in mental healthcare has mainly focused on non‐adherence and specific medications such as antipsychotics [12, 26, 27]. There is little evidence of medication safety experiences in the community post‐discharge, particularly from patient and carer perspectives. This is important as patients with mental illness are known to have different medication safety needs and risks compared to the general population, such as psychotropic polypharmacy and cognitive impairment [6, 16, 28] and multi‐morbidity associated with both physical and mental illness [29, 30].

This study, therefore, aimed to explore stakeholder experiences with and the social processes and practices involved in supporting patients, carers and health professionals with the safe and best use of medicines in the community following a mental health hospital stay.

## 2 Methods

### 2.1 Study Design

This was a qualitative study that drew upon understandings that medication safety activities occur within systems and as social processes and are experienced by patients and carers subjectively within the contexts of their lives [31, 32, 33]. We therefore used semi‐structured interviews to explore care after discharge to support medication‐related activities, the challenges and difficulties with medicine taking after discharge, how people were supported with their medicines, and medicine taking in the community following discharge (see Supplementary File S1).

### 2.2 Setting and Sample

This qualitative study was carried out at a national level in England. People aged 18+ with lived experience of mental illness who had experienced at least one discharge from mental health inpatient care with prescribed medication in the last 5 years, or carers of such individuals, were eligible to take part in the study. The sample additionally included health professionals registered to practise and working in the community in England and Wales, with at least 3 years' experience. Health professionals included those working in specialist community mental health teams (e.g., Home Treatment Teams, Community Mental Health Teams, Early Intervention Teams and drug and alcohol services [34]), general practices, community pharmacy and a small number in secondary care mental health or in teams working across the interface between primary and secondary care. These services are briefly described in Table 1.

**Table 1:** A summary of the different types of community‐based settings [34, 62, 70].

Different types of community‐based care	The support they provide
Community mental health teams (CMHTs)	CMHTs' services focus on providing support for patients with mental illness, particularly those whose needs may not be fully met by general practice. These services also aim to promote recovery and provide ongoing, specialised care.
Crisis team	Crisis teams provide intensive, short‐term support to people experiencing a mental health crisis, aiming to prevent hospital admission whenever possible. However, they are also responsible for facilitating hospital admission if necessary. These teams can offer medication management, ensure co‐ordination with other services for ongoing care, and arrange regular home visits as needed.
Early intervention in psychosis team (EIP)	Early Intervention Teams (EIP) consist of multidisciplinary teams, including care co‐ordinators, psychiatrists, clinical psychologists and other key staff, who support people experiencing a first episode of psychosis. Their aim is to provide the best available treatment, promote recovery and prevent relapse.
Primary care networks (PCNs)	Primary Care Networks (PCNs) are collaborations between GP practices and other health providers, typically covering populations of around 50,000 people. They aim to deliver more personalised and co‐ordinated care by linking general practices with community services, introducing additional services and expanding the multidisciplinary workforce at a local level.
Drug and alcohol service	Drug and Alcohol Services provide interventions for adults with a physical and psychological dependence on alcohol and/or prescribed, over‐the‐counter and illicit substances.
General practice (GP)	General practice is one of the key primary care services in the United Kingdom and often serves as the first point of contact for patients. General practitioners (GPs) manage a wide range of health issues and co‐ordinate care with other healthcare professionals when necessary, including making referrals to other services.
Community Pharmacies	Community pharmacy, also known as retail pharmacy, is an integral part of the NHS primary care system. These pharmacies are highly accessible, often located on high streets, in supermarkets and within neighbourhood centres. They provide a range of services, including essential services such as dispensing medicines and medical appliances, offering health advice, and optional advanced services such as flu vaccinations.

### 2.3 Recruitment and Data Collection

Participants were recruited via social media, professional networks and outreach to third sector, patient and carer groups through emails and online meetings. Interested individuals contacted the research team and received study information by email or post before providing written consent. Semi‐structured interviews were conducted by M.J. remotely online via Zoom/Microsoft Teams to explore medication use and safety post‐discharge. One interview took place face‐to‐face. Interviews were conducted by researcher M.J. between December 2023 and April 2024 and ranged between 23 and 71 min in length, with an average of 50 min. Interviews were recorded via the online platform (or with a digital recorder) and transcribed verbatim. Demographic information was collected verbally from the participant at the end of the interview to allow us to report the diversity of the sample (Tables 2 and 3).

**Table 2:** Summary of recruitment and demographic data.

Demographics by role				
	HCP	Carer	Lived	Total
Age				
18–24	0	0	2	2
25–34	4	0	3	7
35–44	5	1	2	8
45–54	4	0	1	5
55–64	4	2	1	7
65+	0	4	1	5
Sex				
Male	3	2	5	10
Female	14	5	4	23
Non‐binary	0	0	1	1
Gender ID				
Gender same as at birth	17	7	9	33
Gender not same as at birth	0	0	1	1
Ethnicity				
White‐British	7	6	6	19
White‐Irish	1	1	1	3
Asian Indian/British Indian	4	0	0	4
Asian‐Other	1	0	0	1
Asian Pakistani/British Pakistani	1	0	2	3
Black African	1	0	0	1
Mixed‐Other	1	0	1	2
Chinese	1	0	0	1

**Table 3:** Summary of healthcare professionals' roles.

Participant Id	Healthcare professional role	Sector
P6	Senior clinical pharmacist, primary care network	Primary care
P7	Specialist mental health pharmacist	Primary care/secondary care interface
P9	General practitioner	Primary care
P10	General practitioner	Primary care
P11	Nurse—home treatment team	Community team
P12	General practitioner	Primary care
P14	Mental health pharmacist	Secondary care
P17	Nurse	Secondary care
P18	Specialist clinical pharmacist	Primary care
P19	Psychiatrist	Secondary care
P25	Nurse consultant	Secondary care
P31	Advanced clinical practitioner—early intervention team	Community team
P32	Pharmacist—community mental health team	Community team
P33	Pharmacist	Primary care
P35	General practitioner	Primary care
P36	Pharmacist—GP practice	Primary care
P37	Pharmacist—community integrated care	Community team

### 2.4 Analysis

The analysis of the interviews followed a template approach [35, 36]. M.J. led the data analysis, which was conducted iteratively with data collection. In keeping with the template approach [33], the first seven transcripts were first read and re‐read in a process of immersion, and then a coding framework was developed by M.J. from that selection of transcripts and from relevant literature [5, 21, 22, 37, 38]. A selection of transcripts was independently read by R.K. and F.N. (lived experience contributor), and these and the preliminary coding framework were discussed across the research team. As data collection continued, further transcripts were then coded by M.J. using the coding framework using the QSR NVivo® 12 application. Discussion between M.J., R.K. and F.N. of coding and the template coding framework took place at regular intervals as coding proceeded, and the template coding framework was modified and adapted as needed to allow for a rich interpretation of the data. Data collection ceased once saturation was deemed to have been reached and no further new patterns or themes would be interpreted [39]. In a further confirmation exercise, R.K. and F.N. independently read a further 10 transcripts, and these and the final coding framework were further discussed. From these, codes were further organised into sets and interpreted as themes and sub‐themes.

## 3 Results

We conducted 34 interviews with a range of health professionals (*n* = 17) in diverse roles, people with lived experience of mental illness and discharge from inpatient mental healthcare (*n* = 10), and carers of people with lived experience (*n* = 7) (see Tables 2 and 3). Health professionals included seven pharmacists, two nurses/Advanced Clinical Practitioners (ACPs) and four General Practitioners (GPs) in community roles and one pharmacist, two nurses and one doctor working in secondary care (see Table 3). One health professional was working in Wales, with the others working in England.

We identified three main themes and eight sub‐themes (see Table 4), with further details and interview excerpts provided in Supplementary File S2. The main themes were: *(1) Health professionals' collaborative work in the co‐ordination of care and medicines; (2) Fragmentation and lack of continuity of care with medicines for patients and carers; and (3) Patient and carer voice: Shared decisions, information and empowerment*.

**Table 4:** Themes, sub‐themes and codes.

Theme A—Health professional collaborative work in the co‐ordination of care and medicines	
Sub code/Theme	Narrative description
A1. Multidisciplinary healthcare teams and collaborative work	The impact upon medicines safety for patients post mental health discharge of the collaborative working of different multidisciplinary community teams. How these teams co‐ordinated and facilitated medicine activities after discharge and their input into patient care.
A2. Knowledge sharing: Impact upon medication safety	The ways in which knowledge, shared between health professionals, impacted medication safety. This included the difficulties healthcare professionals faced in contacting other teams, for example, between primary and secondary care and the use of technology to give access to and share records.
A3. Discharge plans and medication reviews	What are the processes and plans set out, by health professionals and organisations, to ensure medication safety. What plans for medication review are made, who completes this review and how useful is it?
**Theme B—Fragmentation and lack of continuity of care with medicines for patients and carers**	
B1. Lack of continuity of care and diversion of responsibility	Responsibility is unclear or is diverted and passed between healthcare providers and staff. There is a lack of continuity in care and medicines because the collaborative networks are fragmented. This impacted patients.
B2. Delays for patients and carers	Patients and carers experienced delays in the provision of medicines. There were challenges for patients around the timing and timeliness of prescriptions and dispensing of medicines.
**Theme C—Patient and carer voice: Shared decisions, information and empowerment**	
C1. Shared decisions	Patients and carers were involved in decision‐making in various ways. Sometimes there was choice and shared decision‐making with patients or carers, at other times they were not involved in decisions.
C2. Provision of information to patients and carers	The ways in which patients and carers were given information about their medicines.
C3. Patient and carer empowerment and disempowerment	Variable patient or carer involvement included patients or carers feeling not listened to and disempowered.

### 3.1 Health Professionals' Collaborative Work in the Co‐Ordination of Care and Medicines

#### 3.1.1 Multidisciplinary Healthcare Teams and Collaborative Working

Discharge care and medicines safety were seen to require a co‐ordinated, collaborative and multidisciplinary team approach to provide continuity of care and medication support. Not working collaboratively was considered to have an impact on patient care.

There needs to be a collaborative approach, there needs to be an understanding that we all have a part to play in this, and that we all have equal accountability, and the consequences of not working collaboratively within a team, ultimately does impact patient care.

P14 Pharmacist

Patients talked of co‐ordinated and collaborative approaches from staff being helpful in their post‐discharge care and their medicines. One patient said that since *‘coming home is a huge thing in itself’*, they felt they needed ‘*that wrap around’* approach and ‘*more intensive support’* from healthcare professionals *(P27 Lived experience).* Similarly, for another patient, the collaboration between the GP and the community team was important in making changes to medications.

And in terms of like support, I've been working with my GP, I'm not under the community team. So my GP is my main point of contact. So he organised the switch from the risperidone to the amisulpride and prescribed the fluoxetine. And throughout that process he's liaised with the community team, […] my contact with the community team is through the GP.

P29 Lived experience

A lack of continuity and collaboration was said to impact medications for patients, with staff *‘very hesitant to make changes, especially when someone's just come out of an acute or long‐term mental health admission’ (P33 Pharmacist)*. Participants thought that this could be because the prescribing was outside their competence, though that could therefore leave patients remaining on medicines they were discharged on, which may have been inappropriate.

#### 3.1.2 Knowledge Sharing: Impact Upon Medication Safety

Collaboration involved communication and inter‐professional knowledge sharing across different healthcare sectors and teams. Part of the collaboration between different health professionals involved access to and the sharing of health records across the secondary/primary care interface. ‘*Integrated systems that could share information more seamlessly’* would it was felt provide *‘access to contemporaneous information’ (P32 Pharmacist)*. Communication was similarly said to involve email, letters and access to shared electronic patient records. However, communication was reported to be patchy and variable, with messages not answered, or, as this pharmacist suggested, ‘*sometimes I just feel like it (messaging) just falls on deaf ears’ (P37 Pharmacist).* It was suggested that at times services had not *‘communicated [the] right information, they've not communicated that quickly enough or they've not communicated it correctly’ (P32 Pharmacist).* This GP spoke of how some might be deterred from making efforts to contact other teams to support people in crisis due to difficulties in contacting them and the delays this may cause.

I think for some GPs the temptation may be to say, oh well I'm not going to refer this time to single point of access […] I'm not going to wait 40 min to ring and then get put through to a different department, then it cuts off, then you get put on hold and then you speak to someone who says oh, I'll speak to them tomorrow. That's a lot of work […] but actually because of knowing how risky that cohort is, I will always do that.

P10 GP

Staff commented that electronic medication records were not visible across care boundaries. Therefore, delays or failures in communication could lead to GPs ‘*being left without information about decisions on patients medications’ (P35 GP)*, including what medications had been prescribed, who had prescribed them and any medication plans.

But if we don't have that information [about prescriptions] to know what they're on, is there a plan for it to be up titrated or, you know, when are they going to be seen next. And then obviously with medications like anti‐psychotics, they need appropriate monitoring. So they will need to have, you know, annual blood tests, ECGs [electrocardiograms], check their BMI [body mass index] […] And if we don't know they're on it, then we can't do the appropriate monitoring.

P10 GP

The NHS Discharge Medicines Service (DMS) was established in 2021 to improve communication and the sharing of information around changes to patients' medicines after leaving hospital by referring patients to a named community pharmacy on discharge [40, 41]. This pharmacist spoke of how communication to community pharmacy about medicines had improved with the advent of the DMS.

I remember when DMS came out, discharge medicines service. When that came out so anyone that had been discharged [who were prescribed any] medicine[s] it would go to the community pharmacy as well [as their GP] because previously we [community pharmacy] never saw the discharges. So changes that were made in hospital we'd never see. So that was quite influential and helpful [as] actually community would be aware of the changes that were made in secondary care to the patient's medication.

P18 Pharmacist

#### 3.1.3 Discharge Plans and Medication Reviews

Continuity of medicines could be achieved through clear discharge plans and collaborative processes. A pharmacist working in the community mental health team talked about ongoing planning involving *‘flag(ging) the person when discharge plans are in place’* for ‘*someone who whilst in hospital had been commenced on an antipsychotic depot injection*’ *(P32 Pharmacist)*. However, this nurse described the difficulties that could be encountered with such planning and in providing medication review in the immediate time period after discharge.

…we [community mental health] ask to go to any pre discharge meeting but we're not always invited. It depends on how much forward thinking there has been about the need for home treatment on discharge […] Sometimes we get told on the day of discharge a patient's been discharged. Sometimes we get told after they've gone. After they've gone we can only technically sign up for 48 or 72 h follow up, but a lot of the time patients need a supported discharge, the staff just don't want to tell us that because they've already let the patient go. So when we get there, basically on our first visit, we're just doing a general assessment […] Not everybody will ask about medication, but I would […] so I say what have you been discharged on, how much medication did you get, have you spoken to your GP yet.

P11 Nurse

Care review planning after discharge was described as including goal setting and medication reviews once patients were under community services. One patient spoke of having care co‐ordinators ‘*constantly making sure I was okay with my medication’* and of reviews of medication in ‘*quarterly meetings with the consultant psychiatrist’ (P27 Lived Experience)**.*** Such reviews could be narrow and transactional without consideration for holistic care needs and the co‐development of plans. This was shown in this carer's frustration with the lack of a holistic medication review process for their son.

I'd love for somebody to sit down…. Ten years he's been in, in services, on medication. Surely he deserves for some skilled pharmacist to sit down and properly look at what he's on […] Look at everything and look at his body and his social life and his self‐confidence and look at everything. Because I think they'd change things, I'm sure they would, if they had the time to actually look at everything, but nobody has the time and they just go with what's been done before.

P15 Carer

### 3.2 Fragmentation and Lack of Continuity of Care With Medicines for Patients and Carers

#### 3.2.1 Lack of Continuity of Care and Diversion of Responsibility

Participants reported that there was an impact on patients and carers of fragmentation and disruptions in general care, specifically with care around medicines. This was associated with fragmented collaborative networks and unclear lines of responsibility amongst health professionals, which left patients falling in between ‘*gaps in the spaces between services*’ *(P29 Lived Experience)**.*** Participants talked of a lack of clarity as to who should be allocated to the care of patients once discharged, a situation exacerbated by *‘a lot of silo working’ (P10 GP)*.

I think the challenges for me is continuity of care […] once our patient gets discharged at the key point of discharge, […] who's that patient key contact?[…] Who are they going to link into if there's a problem? […] Is a GP going to do the check? Is the GP or the clinician or the mental health nurse going to support? Who's doing the annual health checks. When did the patient last have medication? Why are they not having it?

P17 Nurse

There was a disconnect between secondary care and general practice as to whether patients had been discharged or not and whether they were attending follow‐ups. This was considered important because non‐engagement with secondary care community services resulted in patients being discharged, but could, in itself, highlight unmet care needs. This GP felt that responsibility was often passed around or diverted with no clear guide as to who should be responsible for patients and their medicines.

…my thoughts about secondary care mental health is that it's very unclear who is responsible for what and it seems it's like a game of tennis. You've got a patient you're worried about and you bat the ball out to someone who you think is going to help. They bat it back and say, no, not our problem, and then you spend ages trying all these different people to fix […].

P9 GP

Further relating to responsibility, the continuity of care was undermined by general practice staff described as ‘*very hesitant to make changes’ (P35 GP)* to medication following discharge. This was said to lead to ‘*quite a bit of toing and froing and the patient is stuck in the middle of it’ (P35 GP)**.***

#### 3.2.2 Delays for Patients and Carers

Patients experienced delays in the provision of medicines in the community following discharge. There were challenges for patients around the timing of prescriptions and the dispensing of medicines. Participants talked of post‐discharge, ‘*there can sometimes be difficulties in the patient obtaining their first prescription’ (P14 Pharmacist)*. One carer talked of delays in the prescription of lithium for the person they cared for, which resulted in a hospital readmission.

So on his discharge he should have lithium and everything. He's sent home from hospital with a supply of lithium. Anyway, because of the change of GPs which has come as a result of being discharged, somewhere in all of this the GP ended up totally missing his lithium off his prescription […]Then when the care home requested more, from what I can gather, the GP wanted a blood test […] Even though it was on the discharge documents, the GP said he needed a blood test […] And in that time, he was sectioned [admitted to hospital under the Mental Health Act] because he'd gone downhill.

P13 Carer

One patient talked of the ‘*stress around (their) communication with the GP and getting the prescription sorted’ (P29 Lived experience)*. Problems could occur because of people being on short‐duration prescriptions and having difficulty if there were delays in the provision of prescriptions and medicines at weekends. This person also talked of mistakes being made with prescriptions that exacerbated these delays and led to further worries.

I'm on weekly [supply of] medications, so it can cause quite a problem if they're sort of delayed, especially over the weekend, 'cause I don't have like a back‐up, you know? So it's kind of like, if they don't give me the medication on the date that I'm asking for is, then I don't have anything to keep me, […] and of course, I do ring up the surgery if they make this mistake […] it causes me a lot of stress and anxiety.

P28 Lived Experience

These difficulties with delays were recognised by health professionals. This pharmacist talked of how delay to important medications was an additional worry for patients already burdened with the changes related to discharge.

I'm aware of some of the urgent nature of some of the bits and for some patients, it's really important for their mental health and for them to feel safe and secure and things like that […] I know the importance of some of these drugs, such as Clozapine […] patients often get quite upset when those bits aren't there, because I think it's just, moving, being transferred from an inpatient setting to a home setting can be difficult enough because there's lots of new challenges […] So sometimes just something as simple as not having your medicines there can just really upset patients.

P37 Pharmacist

### 3.3 Patient and Carer Voice: Shared Decisions, Information and Empowerment

#### 3.3.1 Shared Decisions

There was reported variability in health professionals' perceptions of the extent of patient and carer involvement in decisions about medicines during and following discharge. One health professional talked of *‘a joined [up] approach’*, *‘partnership working’* and *‘putting the patient at the centre of their care’ (P17 Nurse).* A pharmacist said, ‘*patients have to be involved in that choice’ (P14 Pharmacist)*. However, this GP talked about how patients may not be aware of plans for their medications.

…you would hope that they are involved, we talk about shared decision‐making, so you would hope that the plan has been discussed with the service user before discharge. I think it's very variable […] But I think people do talk about feeling that they've been discharged on loads of med[icine]s and they aren't sure what the plan is either.

P12 GP

It was reported that, at times, patients were given a choice, and there was shared decision‐making. But at other times, patients were given responsibility without being involved in decisions.

I've had enough patients contact me to discuss their medications for me to doubt that the patients are involved in those decision‐makings […] I've had enough specifically ethnic minority patients who have been put on a drug but they don't fully understand why, they just have to take it because they don't have a choice in taking it. To know that, I don't think patients are fully being counselled or fully being given options or fully discussing to them why they are being put on a certain drug over another drug.

P35 GP

There was also a balance in how much support was offered and in individualising involvement in decisions after discharge, since ‘*we're all different, we're all individual, so it's a sort of person‐centred approach really, one size will never fit all’ (P2 Carer)*. Similarly, this nurse talked of what person‐centred and individualised approaches might involve.

…always putting the patient at the centre really. And actually, trying to recognise what the challenges are for each individual, you know, so that actually you develop a more person‐centred approach to medication management. Some people don't need any handholding and manage it very well, other people need a great deal of support to understand and get the best from their medicines really.

P25 Nurse

#### 3.3.2 Provision of Information to Patients and Carers

Choice was associated with the provision of information to patients. Information giving was reported to be variable and sometimes limited. For this person with lived experience, information about side effects could be challenging if it were merely presented as a list and not part of a conversation about how side effects might be managed.

Yeah. I mean, like a prime example would be, like a lot of antipsychotics have potential side effects as weight gain, which as someone with an eating disorder is terrifying. […] And it really puts you off, and actually that medication might be the one that really, really helps. But I think what would be really helpful, would be, instead of getting a list of side effects, maybe having a list of a few side effects, but reassurance about them, how they can be treated, like that kind of thing, instead of just saying all negative things about them.

P26 Lived Experience

This ACP talked about information giving being part of trust and that being open and honest about medicines and side effects was an important thing to do.

And I think you've got to be careful because you don't want to scare people half to death with the horror stories of antipsychotic medication, but you also want to be open and honest that these are serious meds and they're not to be sniffed at. […] If you underplay and the client has a negative experience and you've not acknowledged that then, I just think you lose the trust right from the off […] you might not experience these [side effects], but if you do, I accept they're horrendous, and we won't let you tolerate that and we'll think again and we'll do something different.

P31 ACP

#### 3.3.3 Patient and Carer Empowerment or Disempowerment

Patient and carer voice and empowerment was an important aspect of ensuring the best and safe use of medicines during and after discharge. Patients could be empowered or disempowered through their contacts and interactions with healthcare professionals about their medicines post‐discharge. Patients talked of not being listened *to and ‘begging for help’ (P5 Lived Experience).*

And I still struggle, like, on many days, you know, to manage because of the dosage of the medication. And I have brought this across many times, you know, with my team, my CPN [community psychiatric nurse]. I've told them many times but I think the problem with all these experiences that … they do not want to listen to me. Like, I try, I try, I try, I explain my situation, even with my mental health advocate. But they don't really pay any attention to what I'm explaining to them or they're not listening to me.

P30 Lived Experience

Patients could be left disempowered. As one Pharmacist said, of patients on admission, that staff *‘take complete ownership of their medicines’* and ‘*you take that empowerment away from them’* and that then at discharge patients were left to fend for themselves because ‘*all of a sudden, you're given this bag of drugs, and say, okay, off you go now, off you go home, and you just carry on doing what you're doing’ (P14 Pharmacist)**.*** Patients talked of wanting to be involved in decisions about medicines after discharge, without being patronised, and *‘to be treated like I'm not stupid’* (*P28 Lived Experience)**.*** There was, however, a sense from people with lived experience that if they asked questions or challenged decisions, they might be seen as ‘difficult patients’.

One thing that I really wanted to avoid was I didn't want to be seen as like a difficult patient. I didn't want to be so insistent on switching that then I became seen as, I don't know, someone who's…. I don't know. I just get the impression that if you're seen as a demanding patient or as a patient who thinks they know best or something, that that can kind of impact how you're seen clinically I think. So I wanted to avoid that. I didn't want to end up being kind of perceived as someone who's hard to work with.

P29 Lived Experience

## 4 Discussion

### 4.1 Summary of Key Findings

Our study highlights that care and medication safety after inpatient mental health stays often lack co‐ordination between patients, carers and services. Participants described poor communication—especially between secondary and community care—and noted that time pressures, disconnected record systems and limited information sharing with GPs contributed to unclear care planning. Continuity of care was disrupted due to unclear responsibilities for patient review and follow‐up, causing service disconnects and patients falling through gaps. Participants reported delays in prescribing, providing and reviewing medications, with inconsistent information given to patients. Patient and carer voices were often unheard, leaving them disempowered, and shared decision‐making was unclear—sometimes involving patients, other times placing responsibility on them without their input.

### 4.2 Organisation and Continuity of Care

Our findings here have illuminated the need within the NHS for more defined strategies to integrate services and clearer organisation of care concerning mental health discharge and follow‐up care, particularly between specialist mental health services and general practice. Few studies have focused upon broader medication safety issues beyond non‐adherence [12], such as those explored in our study, including delays to prescriptions and supply and a lack of planning. Similarly to the findings here, Ayre et al. [42] reported poor continuity of follow‐up and communication gaps between siloed health teams in community mental healthcare. Participants in our study also noted that fragmented patient record systems hindered communication, causing delays in discharge summaries and prescriptions. Such challenges with information sharing and digital record integration are well documented in the literature, along with their impact on medication safety [43, 44].

Pharmacist involvement has been suggested to improve care co‐ordination and patient knowledge and provide appropriate injectable antipsychotic medication counselling, including in discharge planning [45, 46, 47]. However, a review of 45 interventions to improve discharge from inpatient mental healthcare identified limited pharmacist involvement, and where there was pharmacist involvement, it tended to be to address patient adherence and not wider medication safety issues [12]. Whilst community pharmacy staff did not take part in our study, the DMS [40] was discussed as a means to improve communication around medicines across care interfaces. In addition, the New Medicines Service (NMS), which aims to support people to learn more with their community pharmacist about specific medicines that have been newly prescribed during hospital admission, has been seen as a cost‐effective service for delivering improved medication safety outcomes [48, 49] with the current revised service model of the NHS Community Pharmacy in England includes depression as a therapeutic area within the NMS. This may allow community pharmacists to support people in managing their medicines through enhanced integration of local care pathways, signposting and data sharing [48]. It has been suggested that there is a need for community pharmacists to be more involved in the provision of mental health services [50, 51, 52, 53], and evidence suggests they may effectively deliver medication support to people with depression and anxiety [54].

### 4.3 Empowerment and Disempowerment

A key finding of our study was how patients and carers expressed feelings of being unheard and disempowered. Although all groups valued shared decision‐making, patients and carers felt only minimally involved in decisions about their medicines. Collaborative decision‐making, which includes both patient and clinician expertise, is widely recognised to improve clinical outcomes and patient satisfaction [55, 56, 57, 58]. Shared decision‐making also fits into models of person‐centred care, which aims to meet patients' needs and place the patient narratives at the centre [59, 60] and has been emphasised and valued in NHS policy [61, 62, 63]. Previously, interactions between patients and community pharmacists in the context of medicines have been seen as dynamic, variegated and fragile and requiring a more personalised care approach [63]. In a survey study in mental health settings, Pappa et al. [55] found a disparity between clinicians' perceptions of shared decision‐making and patients, with clinicians being far more positive about their collaborations than patients. Pappa et al. found a lack of consistency in shared decisions and that shared decision‐making was hampered by a lack of time, information and decision‐making tools. Ayre et al., in a multimethod study seeking to understand medication safety for patients with mental illness in primary care, found power imbalances between healthcare professionals and patients, a lack of involvement in shared decision‐making or open conversations about therapeutic options [42]. Reasons for discontinuing mental health medications have included a lack of dialogue and being left out of decisions involving treatment [64, 65]. Shared decision‐making in mental health medication is evolving from medicine‐focused consultations to building long‐term relationships and tailoring treatment to individual needs, lifestyles and social functioning [66]. In developing models of shared care, it has previously been suggested that importing models from physical health is problematic, particularly since patients with mental health issues can feel disempowered [67]. Fox [68], in an autoethnography, discussed shared decision‐making from a lived experience perspective and concluded that active participation in underpinning approaches to mental health policy care and treatment was important. Fox made a contrast between biomedical models that focused upon evidence‐based treatments and recovery models that viewed patients as experts in their own care [68].

### 4.4 Implications for Policy and Practice

This study raises a number of implications for policy and practice. Developing greater continuity of care has already been set out by others [42, 69] and is detailed in policy [61, 62, 70]. We suggest that care continuity needs clearer pathways and defined responsibilities, organised around collaborative working. Responsibility for patients' medication was often unclear, causing delays and forcing patients and carers to find their own solutions. Effective communication and system integration are essential for collaboration, especially across care sectors [43].

In a recent review of mental and general health discharge interventions, Tyler et al. found that low‐ and medium‐complexity discharge interventions are more effective at reducing readmissions than multicomponent interventions [71]. This suggests interventions should be simple enough for proper implementation but still address multiple safety issues linked to discharge [71]. In planning future models of care, the involvement of community partners, people with lived experience and carers in policy planning has been advocated and seen as one of the core components of collaborative care [58, 68, 70]. We recommend involving stakeholders in policy development—including care continuity pathways—and focusing on person‐centred care models that promote shared decision‐making, include medication management and information, and empower patients in their choices.

### 4.5 Strengths and Limitations

A key strength of this paper is its inclusion of diverse lived experiences, carer voices and health professionals, allowing for multiple perspectives on medications and follow‐up care with more transferable findings. Having a research team member with lived experience at all stages of the research was valuable in providing clear feedback on recruitment materials, positive feedback on data collection and notably enriched the analysis by providing a different perspective (see Supplementary File S3). Our study advisory group, which included a person with lived experience and a carer, was able to reassure the research team that the project was on track and appropriate in its approach. Additionally, the study advisory group helped to troubleshoot recruitment challenges by supporting the team to identify new avenues.

The team's interdisciplinary backgrounds in pharmacy, psychology, social care and health services research enriched the study through diverse perspectives on medicines safety and care continuity. While this was a strength, it may also have introduced interpretive bias, with a stronger focus on system‐level processes. To enhance reflexivity, the team held regular discussions and maintained reflexive notes throughout data collection and analysis to consider how their roles and assumptions might have shaped interpretation.

Although this was a national study, most participants were from North‐West England, limiting transferability to other regions. Health professionals were mainly recruited via researchers' networks, and recruiting carers and people with lived experience through social media meant we could not verify if all met the inclusion criteria. We also introduced measures to confirm participants were from the United Kingdom and met our inclusion criteria as described elsewhere [72, 73]. Both using social media and online interviews to recruit may have marginalised people from some lower socio‐economic or older age groups because of access to digital tools, but conversely, may have encouraged more younger people to take part. Our sample did have an element of diversity across different ages and ethnicities (Table 2).

## 5 Conclusions

This study highlights key medication safety challenges during transitions from inpatient mental healthcare to primary care, including fragmented communication, unclear post‐discharge medication responsibilities, and inconsistent patient and carer involvement. These issues led to delays, inadequate reviews and poor care continuity, raising the risk of errors and harm. Improving medication safety requires clearer care pathways, better collaboration and active patient and carer involvement in shared decision‐making.

## Author Contributions

**Mark Jeffries:** funding acquisition, conceptualisation, writing – original draft, writing – review and editing, methodology, investigation, formal analysis, validation. **Fiona Naylor:** writing – review and editing, methodology, formal analysis, validation. **Natasha Tyler:** funding acquisition, conceptualisation, writing – review and editing, validation. **Catherine Robinson:** funding acquisition, conceptualisation, writing – review and editing, validation. **Maria Panagioti:** funding acquisition, conceptualisation, writing – review and editing, validation. **Richard N. Keers:** funding acquisition, conceptualisation, writing – review and editing, methodology, formal analysis, supervision, validation.

## Disclosure

The views expressed are those of the authors alone and not necessarily those of the NIHR, or the Department of Health and Social Care.

## Ethics Statement

This study was approved by the University of Manchester Research Ethics Committee 2 (ref: 2023‐17915‐32081). As this was a national‐level study not requiring recruitment through a specific NHS organisation, Health Research Authority (HRA) approval was not required.

## Consent

Written informed consent was obtained from all participants. All interview extracts are fully anonymised. Fiona Naylor gave her consent for her comments to be used in Supplementary File S3.

## Conflicts of Interest

The authors declare no conflicts of interest.

## Supporting information

Supplementary_File_S1_Interview_Topic_Guide.

Supplementary_File_S2_Main_Themes_Sub_Themes_and_Extracts.

Supplementary_File_S3_Reporting_Framework_on_lived_experience_engagement.

## Data Availability

This is a qualitative study confined to relatively small groups of healthcare professionals in specific roles, people with lived experience and carers. Making the full transcripts publicly available could therefore potentially lead to the identification of participants. Our ethics approval was granted based on the anonymity of the individuals consenting to participate and specifically referred to only anonymised quotations being used in reports. As such, participants did not fully consent to their transcripts being made publicly available. Therefore, full transcripts cannot be made publicly available; however, detailed themes and full extracts from transcripts are available in a Supplementary File.
