# Supplementary material for: Exploring Medicines Optimisation and Safety in the Community Following Mental Health Hospital Discharge: A Qualitative Interview Study
Source: Health Expect. 2025 Dec 25;29(1):e70535. doi: 10.1111/hex.70535 (PMC12741015; doi:10.1111/hex.70535)
Supplement: Supplementary file 1 — Supplementary_File_S1_Interview_Topic_Guide. [file HEX-29-e70535-s002.docx]

**Developing a framework for medication optimisation and safety in primary care following mental health hospital discharge.**

**Interview Topic guides - Part A – People with lived experience only**

***Interviewer introduction:***

My name is…………….I am researcher at the University of XXXXXXXXX. We are carrying out a study looking at how medicines might be used safely and in the best way by patients with mental illness and their carers in the community after they have been discharged from mental health hospital. We would like to find out the views of patients who have had a stay in a mental health hospital during the last few years and were receiving medication for their mental health in the community after they were discharged.

This interview will last for about 45 minutes. During this time, I’d like to discuss your experiences of your care after leaving the hospital particularly focusing on your medicines and any support you needed or got with your medicine taking. We will also together complete a diagram to show what your medication support ‘looked like’.

- If you agree, I would like to record our interview. This will make sure I have precise details of what we discuss.
- No-one will be able to identify you in any reports containing quotes from your interview.
- Direct responses from your interview may be used in a future workshop for this study but no one will be able to identify you from these.
- Please try to avoid naming locations and staff specifically. Any written report will have any identifiable information (names/locations) removed.
- You may stop the recording at any time or take a break
- If the interview is on ZOOM/TEAMS you may have your camera switched off if you wish.
- If you are in a group interview we will need to conduct and record this for everyone in the interview in the same way – either on ZOOM/TEAMS with everyone on camera or everyone with their camera switched off or over the telephone. If that is not possible we will interview you separately not in the group.

Any discussions that take place during the interview are confidential. If, during the interview, we have concerns about your safety or the safety of others, we may ask you to contact an appropriate person about this, such as your care team, other health professional or a family member. If we consider it to be serious, we may have to report this. In no other circumstances will we discuss with, your doctors, care team or other health professional what we have talked about in the interview or contact them on your behalf.

Unless you have any questions for me, I will start the recording and we can begin.

| **Main Focus** | **Questions** | **Probes and Prompts** |
| --- | --- | --- |
| **Medication activities - Key facilitators - What helps?** | 1. Thinking about a hospital stay you had in the last five years and about the medicines you were prescribed after you left the hospital, can you tell me about what happened with them?  2. Can you tell me what helped with your medicine taking after being discharged? Did that help you feel safe?  3. What do you feel was needed to help you take your medicines during the time after discharge?  4. During the time after you left hospital how involved in any decisions about your medicines did you feel? | Did your medicines change much after discharge? What were the biggest difficulties? What were the problems? How can they be resolved? Have any changes been explained?  What are the things that helped? What helped in getting prescriptions? Why? Did you have any reviews of your medications? If so how, by whom, how did they help?  Practical help? Support? Information? What sort of support?  Did you feel supported to take your medicines safely?”  Did you think there was a plan for your medicines? In what ways were you involved in that plan? Do you want to be involved? Who makes the decisions about your medicines? What most helps with that? |
| **Support - Information and knowledge that has been shared about medicines** | 5. Thinking about medicines you were given to take after you left hospital, or were prescribed soon after you left. Can you talk be through the support you’ve had in terms of any information you were or have been given/conversations you had? | Who has talked to you? Helpful/unhelpful? What kind of information did you get (e.g. written, verbal)? How do you prefer to get information? Timing? Too much or too little? What helps and what doesn’t help? Did/do you have to ask or prompt people to give you information? How do you feel about the conversations you’ve had? Has anyone helped you get support? |
| **Support – People who help with needs, provision** | 6. I want to you to think about who supported you with your medicines after you were discharged from the hospital. This diagram has you in the middle and each circle represents support you got. So the first circle would be people who may have given you most support and the outer one those people who might help but not as much. Can we, together add some people in. | Work through the HMT allowing the participant to add people. During and at the end – ask why each person is where they are and in what ways they help, the support they provide, why that matters. |
| **Key Challenges – Problems and difficulties** | 6. After you went home from hospital do you think there were any issues with your medicines that might have made you feel less safe? Why do you think they happened?  7. What do you think can be put in place to help you feel more safe in future if you or other people were discharged from hospital again? | Were there any mistakes? Were there things you weren’t sure of, or needed help with?  How do you think it would help? |

**Interviewer conclusions:**

Is there anything you would like to add?

Is there anything you think we have missed?

Is there anything you’d like to go back over?

Many thanks for taking the time to help us with this study. Your contribution has been extremely valuable. If you wish you can request to receive a summary of the findings of this research study, just ask us, using the contact details on the information sheet. In the meantime please feel free to contact either myself or the other researcher(s) involved if you have questions in future.

| Demographic Data (Lived Experience) | 2. Age: | 3. Sex: | 4. Gender ID | 5. Ethnicity: |  |
| --- | --- | --- | --- | --- | --- |
| All information will be collected and stored on a secure research storage drive and treated in the strictest confidence.  We are only collecting this information to ensure we have a diverse sample   1. Geographical Area.-   Where do you live (County or City)_  _______________ | 18 – 24 ☐  25 – 34 ☐  35 – 44 ☐  45 – 54 ☐  55 – 64 ☐  65+ ☐ | Male ☐  Female ☐  Prefer not to say ☐  : | Is your gender the same as that assigned to you at birth?  YES ☐  NO ☐ | White British ☐  White Irish ☐  White Any Other Background ☐  Mixed White/Black Caribbean ☐  Mixed White/Black African ☐  Mixed White/Asian ☐  Mixed Mixed Any Other Background ☐  Asian Indian ☐  Asian Pakistani ☐ | Asian Bangladeshi ☐  Asian Any Other Background ☐  Black Caribbean ☐  Black African ☐  Black Any Other Background ☐  Other Chinese ☐  Other Any Other – please state  Other Prefer not to say ☐ |
|  |  |  |  |  |  |

**Developing a framework for medication optimisation and safety in primary care following discharge from mental health hospitals**

**Interview Topic guides**

**Part B – Carers only**

***Interviewer introduction:***

My name is…………….I am researcher at the University of XXXXXXXX. We are carrying out a study looking at how medicines might be used safely and in the best way by patients with mental illness and their carers in the community after they have been discharged from mental health hospital. We would like to find out the views of the carers of patients who have had a stay in a hospital during the last few years, and were receiving medication for their mental health in the community after they were discharged.

This interview will last for about 45 minutes. During this time, I’d like to discuss your experiences as a carer of someone who has previously had inpatient mental health care particularly focusing on their medicines and any support they needed or got with their medicine taking.

- If you agree, I would like to record our interview. This will make sure I have precise details of what we discuss.
- You may stop the recording at any time or take a break
- No-one will be able to identify you in any reports containing quotes from your interview.
- Direct responses from your interview may be used in a future workshop for this study but no one will be able to identify you from these.
- If the interview is on ZOOM/TEAMS you may have your camera switched off if you wish.
- If you are in a group interview we will need to conduct and record this for everyone in the interview in the same way – either on ZOOM/TEAMS with everyone on camera or everyone with their camera switched off or over the telephone. If that is not possible we will interview you separately not in the group.
- Please try to avoid naming locations and staff specifically. Any written report will have any identifiable information (names/locations) removed.

Any discussions that take place during the interview are confidential. If, during the interview, we have concerns about your safety or the safety of others, we may ask you to contact an appropriate person about this, such as a health professional or a family member. If we consider it to be serious, we may have to report this. In no other circumstances will we discuss with, the doctors, care team or other health professionals of the person you care for what we have talked about in the interview or contact them on your behalf.

Unless you have any questions for me, I will start the recording and we can begin.

| **Main Focus** | **Questions** | **Probes and Prompts** |
| --- | --- | --- |
| **Medication activities - Key facilitators - What helps?** | 1. Thinking about about a hospital stay the person you care for had in the last five years and the medicines they were prescribed after they left hospital, can you tell me about what you think helps with their medicine taking ?  2. What do you feel is needed to help the person you care for take their medicines and stay safe?  3. Think about the decisions about the medicines for the person you care for after they left hospital. Who was involved in those decisions, and how were they made? | What are the things that help? What helps in getting prescriptions? Why? As far as you know, has the person you care for had any reviews of their medications? If so how, by whom, how did they help? What part have you played in that? Do you talk about these reviews with the person you care for?  Practical help? Support? Information? What sort of support?  Do you think there’s a plan for their medicines? In what ways have they been involved in that plan? Have you been involved? Do you want to be involved? Who makes the decisions about your medicines? What most helps with that? |
| **Support - Information and knowledge that has been shared about medicines** | 4. Thinking about medicines the person you care for were given to take after they left hospital, or have been prescribed soon after they left. Can you talk me through the support you think they’ve (and or yourself) had in terms of information you or they were or have been given/conversations you had? | Who has talked to you/them? Helpful/unhelpful? Did you/they get any written information? How do you /they prefer to get information? Timing? Too much or too little? What helps and what doesn’t help? Did/do you/they have to ask or prompt people to give you information? How do you/they feel about asking questions? How do you feel about the conversations you’ve had? |
| **Support – People who help with needs, provision** | 5. I want to you to think about who supported the person you care for with their medicines after they left hospital. Can you tell me about how they are supported by yourself and others with their medicines?  6. I want you to think about whether **YOU** were supported to help the person you care for with their with medicines after they were discharged from the hospital. This diagram has you in the middle and each circle represents support you got. So the first circle would be people who may have given you most support and the outer one those people who might help but not as much. Can we, together add some people in. | What support do you offer them? In what ways do you think that helps? Who are the other people who are involved in supporting them with their medicines? How do they help?  Work through the HMT allowing the participant to add people. During and at the end – ask why each person is where they are and in what ways they help, the support they provide, why that matters. |
| **Key Challenges – Problems and difficulties** | 7. After the person you care for went home from hospital do you think there were any issues with their medicines that might have made them feel less safe?  8. What do you feel are the challenges around their medicines? | What do you think can be put in place to help them feel more safe? What helps people stay safe?  Have there been any changes to their medicines? Have those changes been explained to you? To them??  What are the biggest difficulties? What are the problems? How can they be resolved? |

**Interviewer conclusions:**

Is there anything you would like to add?

Is there anything you think we have missed?

Is there anything you’d like to go back over?

Many thanks for taking the time to help us with this study. Your contribution has been extremely valuable. If you wish you can request to receive a summary of the findings of this research study, just ask us. In the meantime please feel free to contact either myself or the other researcher(s) involved if you have questions in future.

| Demographic Data (Carers) | 2. Age: | 3. Sex: | 4. Gender ID | 5. Ethnicity: |  |
| --- | --- | --- | --- | --- | --- |
| All information will be collected and stored on a secure research storage drive and treated in the strictest confidence.  We are only collecting this information to ensure we have a diverse sample   1. Geographical Area.-   Where do you live (County or City)_  _______________ | 18 – 24 ☐  25 – 34 ☐  35 – 44 ☐  45 – 54 ☐  55 – 64 ☐  65+ ☐ | Male ☐  Female ☐  Prefer not to say ☐  : | Is your gender the same as that assigned to you at birth?  YES ☐  NO ☐ | White British ☐  White Irish ☐  White Any Other Background ☐  Mixed White/Black Caribbean ☐  Mixed White/Black African ☐  Mixed White/Asian ☐  Mixed Mixed Any Other Background ☐  Asian Indian ☐  Asian Pakistani ☐ | Asian Bangladeshi ☐  Asian Any Other Background ☐  Black Caribbean ☐  Black African ☐  Black Any Other Background ☐  Other Chinese ☐  Other Any Other – please state  Other Prefer not to say ☐ |
|  |  |  |  |  |  |

.

**Developing a framework for medication optimisation and safety in primary care following discharge from mental health hospitals**

**Interview Topic guides -Part C - Health Professionals**

***Interviewer introduction:***

My name is…………….I am researcher at the University of XXXXXXXX. We are carrying out a study looking at medication safety processes in the community for patients and their carers who has been discharged from inpatient mental health care. We would like to find out the views of the health professionals involved in the care of patients and their carers in the community who have been recently discharged from inpatient mental health care and are receiving medication support..

This interview will last for about 45 minutes. During this time, I’d like to discuss your experiences as a health professional involved in the care provision in the community for people and their carers who have previously had inpatient mental health care particularly focusing on their medicines and the support they and you as a health professional may need and get with medication related issues.

- If you agree, I would like to record our interview. This will make sure I have precise details of what we discuss.
- You may stop the recording at any time or take a break
- No-one will be able to identify you in any reports containing quotes from your interview.
- Direct responses from your interview may be used in a future workshop for this study but no one will be able to identify you from these.
- If the interview is on ZOOM/TEAMS you may have your camera switched off if you wish.
- If you are in a group interview we will need to conduct and record this for everyone in the interview in the same way – either on ZOOM/TEAMS with everyone on camera or everyone with their camera switched off or over the telephone. If that is not possible we will interview you separately not in the group.
- Please try to avoid naming locations and staff specifically. Any written report will have any identifiable information (names/locations) removed.

Any discussions that take place during the study are confidential. If, during the interview, we have concerns about your safety or the safety of others, we may ask you to contact an appropriate person about this, such as your line manager. If we consider it to be serious, we may have to report this. In no other circumstances will we discuss with anyone else, outside of the research team, what we talk about in the interview.

Unless you have any questions for me, I will start the recording and we can begin.

| **Main Focus** | **Questions** | **Probes and Prompts** |
| --- | --- | --- |
| **Medication safety activities and practice - Key facilitators - What helps?** | 1. Thinking about people you care for in the community who have had a recent inpatient mental health hospital stay within the last five years and their prescribed medicines. Can you talk me through what may typically happen in the time period after discharge?  2. What happens to help people take their medicines and stay safe after mental health hospital discharge?  3.  Thinking about any decisions that are needed to be made in the community about the medicines for patients in the time period after hospital discharge. How are those decisions made and how are plans put together? | What happens? What is your involvement? What is the involvement of others and services? What happens immediately after discharge? Medicines -meds rec, adjustments stopping/starting.  What does ongoing care look like? What sort of follow up is there for people who have left inpatient care? What is the purpose of follow-up? How do you feel this helps?  Who makes the decisions about patients medicines? What do you feel is important about decision making? What most helps with that? Are specific individual plans made for patients’ medication? Who is involved in the plan (incl. patients and carers)? Does having a plan help? If so in what ways? If not why? |
| **Support - Information and knowledge that has been shared about medicines** | 4. Thinking about people in the community who have had a recent inpatient mental health hospital stay and their medicines. What sort of information around medicines is provided?  5. Do you think that you or other health care professionals need information about medicines prescribed to this patient group after discharge? What sort of information do you need, is this provided and how is this provided? | How well informed do you think patients/carers and other health professionals are? What is the process for knowledge transfer/giving information to patients or other health professionals? How does that work? What resources are needed? What are the barriers and facilitators to information provision?  Do you think that patients/carers need information about medicines they are prescribed? What sort of information do they need, and if and how do they receive it? |
| **Support – provision** | 6. I want to think about social networks available for people who leave in patient care might get? What sort of support is there? How does it help?  7. I want you to think about whether **YOU** are supported to help the people you care for with their with medicines after they were discharged from hospital. This diagram has you in the middle and each circle represents support you got. So the first circle would be people who may have given you most support and the outer one those people who might help but not as much. Can we, together add some people in. | What support do people get? In what ways do you think that helps? Who are the people who are involved in supporting them with their medicines? How do they help? Is this important? Why?  Work through the HMT allowing the participant to add people. During and at the end – ask why each person is where they are and in what ways they help, the support they provide, why that matters. |
| **Key Challenges – Problems and difficulties** | 8. After patients are discharged from mental health hospital do you think there are any challenges with their medicines that might lead to safety concerns? Can you describe these challenges, and why you think they happened?  9. What do you think can be put in place to help you, other health professionals and patients/carers feel safer with medicines in future after discharge from a mental health hospital? | Who might be involved, why might they occur, how often do they occur  Are there separate things that could be done to support HCPs, and patients/carers? Why have you suggested these things? |

**Interviewer conclusions:**

Is there anything you would like to add?

Is there anything you think we have missed?

Is there anything you’d like to go back over?

Many thanks for taking the time to help us with this study. Your contribution has been extremely valuable. If you wish you can request to receive a summary of the findings of this research study, just ask us. In the meantime please feel free to contact either myself or the other researcher(s) involved if you have questions in future.

| Demographic Data (Health Professionals) | 2. Role | 3. Age: | 4. Sex: | 5. Gender ID | 6. Ethnicity: |  |
| --- | --- | --- | --- | --- | --- | --- |
| All information will be collected and stored on a secure research storage drive and treated in the strictest confidence.  We are only collecting this information to ensure we have a diverse sample   1. Geographical Area.-   Where do you work (County or City)_  _______________ | Occupation and community setting they work in | 18 – 24 ☐  25 – 34 ☐  35 – 44 ☐  45 – 54 ☐  55 – 64 ☐  65+ ☐ | Male ☐  Female ☐  Prefer not to say ☐  : | Is your gender the same as that assigned to you at birth?  YES ☐  NO ☐ | White British ☐  White Irish ☐  White Any Other Background ☐  Mixed White/Black Caribbean ☐  Mixed White/Black African ☐  Mixed White/Asian ☐  Mixed Mixed Any Other Background ☐  Asian Indian ☐  Asian Pakistani ☐ | Asian Bangladeshi ☐  Asian Any Other Background ☐  Black Caribbean ☐  Black African ☐  Black Any Other Background ☐  Other Chinese ☐  Other Any Other – please state  Other Prefer not to say ☐ |
