# Supplementary material for: Exploring Medicines Optimisation and Safety in the Community Following Mental Health Hospital Discharge: A Qualitative Interview Study
Source: Health Expect. 2025 Dec 25;29(1):e70535. doi: 10.1111/hex.70535 (PMC12741015; doi:10.1111/hex.70535)
Supplement: Supplementary file 2 — Supplementary_File_S2_Main_Themes_Sub_Themes_and_Extracts. [file HEX-29-e70535-s001.docx]

**Exploring Medicines Optimisation and Safety in the Community Following Mental Health Hospital Discharge: A Qualitative Interview Study**

**Supplementary File - S2. Detailed Themes, Sub Themes and interview excerpts.**

| **Theme A – Health professional collaborative work in the coordination of care and medicines** | |
| --- | --- |
| **Sub code/Theme** | **Narrative description** |
| A1. Multidisciplinary healthcare teams and collaborative working | The impact upon medicines safety for patients post mental health discharge of the collaborative working of different multidisciplinary community teams. How these teams co-ordinated and facilitated medicine activities after discharge and their input into patient care. |
| A2. Knowledge sharing: Impact upon medication safety | The ways in which knowledge, shared between health professionals, impacted upon medication safety. This included the difficulties health care professionals faced in contacting other teams for example between primary and secondary care and the use of technology to give access to and share records. |
| A3. Discharge plans and medication reviews | What are the processes and plans set out, by health professionals and organisations, to ensure medication safety. What plans for medication review are made, who completes this review and how useful is it? |
| **Theme B –** **Fragmentation and lack of continuity of care with medicines for patients and carers** | |
| **Sub code/Theme** | **Narrative description** |
| B1. Lack of continuity of care and diversion of responsibility | Responsibility is unclear or is diverted and passed between healthcare providers and staff. There is a lack of continuity in care and medicines because the collaborative networks are fragmented. This impacted upon patients. |
| B2. Delays for patients and carers | Patients and carers experienced delays in the provision of medicines. There were challenges for patients around the timing and timeliness of prescriptions and dispensing of medicines. |
| **Theme C - Patient and carer voice: Shared decisions, information and empowerment.** | |
| **Sub code/Theme** | **Narrative description** |
| C1. Shared decisions | Patients and carers were involved in decision making in variable ways .Sometimes there was choice and shared decision making with patients or carers, at other times they were not involved in decisions. |
| C2. Provision of information to patients and carers | The ways in which patients and carers were given information about their medicines. |
| C3. Patient and carer empowerment or disempowerment | Variable patient or carer involvement included patients or carers feeling not listened to and disempowered. |

**Theme A – Health professional collaborative work in the coordination of care and medicines**

| **Sub code/Theme** | **Narrative description** |
| --- | --- |
| A1. Multidisciplinary healthcare teams and collaborative working | The impact upon medicines safety for patients post mental health discharge of the collaborative working of different multidisciplinary community teams. How these teams co-ordinated and facilitated medicine activities after discharge and their input into patient care. |
| There’s a lot of silo working sometimes. You know, working in silos, in that the GPs, we will have our guidelines about what’s appropriate and we’ll have our idea about what is safe and what is not safe, I guess, and the risks, you know, if we’ve ever got any doubt that this person is not going to keep themselves safe, then they should be, you know, reassessed, whether that means readmission, that’s not our decision. I’ve never been involved with a sectioning or anything like that, so that would be a separate interest, I guess. ***P10GP***  There needs to be a collaborative approach, there needs to be an understanding that we all have a part to play in this, and that we all have equal accountability, and the consequences of not working collaboratively within a team, ultimately does impact patient care. And I think what people forget is around the costing for inpatient services, and actually getting it right on discharge will potentially prevent that readmission. And, you know, people that are constantly readmitted, they are going to end up losing their job, potentially losing where they live, losing relations, you know, so that cost, you can’t monetize that cost, that cost to life is significant. ***P14 Pharmacist***  I think, as we said before, obviously having had been an inpatient myself I think coming home is a huge thing in itself. Obviously you’ve been institutionalised so to speak and I think you need that wrap around. I think obviously carers are imperative, but also for me, like I said, I think something like the early intervention team or something similar. I know obviously there’s the crisis team, there’s the home treatment team, so I think someone should definitely be under some form of that. I don’t feel really like the GP has enough resources and time to manage that. I think you need more intensive support. ***P27 Lived Experience***  They’re a really vital part in our, sort of, set up we work very, very closely with our community pharmacies. And they’re often the ones that will flag up concerns about people to us really*.* ***P25 Nurse***  And in terms of like support, I've been working with my GP, I'm not under the community team. So my GP is my main point of contact. So he organised the switch from the risperidone to the amisulpride and prescribed the fluoxetine. And throughout that process he's liaised with the community team, but they've…my contact with the community team is through the GP, rather than being on the… ***P29 Lived Experience***  Exactly that because there can be delays. We’ve had it from both ends, where our inpatient services have either not communicated right information, they’ve not communicated that quickly enough or they’ve not communicated it correctly […] So I work on the community side now, so I’m a pharmacist in our community mental health team, and I’m often the conduit to sorting out the problem. So our community mental health team have got quite good processes in place, so I gather it’s probably a more nationally mandated thing in that everyone has their 72 hour follow-up after discharge from hospital because of the risks associated with an inpatient discharge from mental health services. And I don’t know if it’s because I work in older adult services, I would normally say CPNs but we’ve actually got non nurses who fulfil that sort of care coordinator role as well, but they are pretty good at checking up on medicines because older people tend to be on more things, and actually they’ve learned that it’s, not more often than not but it’s not uncommon for there to be discrepancies that need adjusted within that. So they’re good at identifying that there’s discrepancies and they’ll often come to me for support to try and resolve it because they haven’t necessarily established where the discrepancy has arisen from, and contacting or figuring out who the right people or person is to speak to to try and get that sorted out as soon as possible. ***P32 Pharmacist*** | |
| A2. Knowledge sharing: Impact upon medication safety | The ways in which knowledge, shared between health professionals, impacted upon medication safety. This included the difficulties health care professionals faced in contacting other teams for example between primary and secondary care and the use of technology to give access to and share records. |
| Sometimes they’ll come to the front desk with a, you know, a pink slip which is a carbon copy of what has been prescribed, you know, as they left. So they may have been given a couple of days’ worth of supply and then told GP to continue. But if we don’t have that information to know what they’re on, is there a plan for it to be up titrated or, you know, when are they going to be seen next. And then obviously with medications like anti-psychotics, they need appropriate monitoring. So they will need to have, you know, annual blood tests, ECGs, check their BMI, you know, things like that. And have a discussion with how they’re getting on. And if we don’t know they’re on it, then we can’t do the appropriate monitoring. ***P10 GP***    Depends on the ward, to be honest, how much information they’re sending. If the medication is going to be prescribed by the GP the care coordinator at the CMHT if the patient has one probably isn’t going to be told what they’re discharged on. I was a care co for nearly 21 years. I was never told what the patient had been discharged on, I had to ask the patient. And even when we moved to computerised systems that information is not necessarily documented. Don’t get me wrong, some doctors will, but because the discharge notification can take days and days to come on the system by the time it’s on the system the patient would usually run out of the medication. It’s not been inputted on the day, and what they have to input isn’t specific. ***P11 Nurse***    Again, you’re still relying on…but that just shows other (NHS Trust) staff what’s been prescribed for the patient. The GP wouldn't be able to see that. Within (NHS Trust), our (name of EMR) has something called (city) care record, which may have been mentioned already. That allows us to access a GP record. We cannot access everything. Generally speaking what we can see is things like current medication, previous medication, significant test results and diagnosis and allergies. In all honesty it’s not our business to see much more than that I wouldn’t have said. Now, when I went to (place), anybody who is on (name of EMR) can access the (city) care record. You have to click on a button to say that you’ve got permission, et cetera, but it’s that basic… Now, I don’t know what (place) has now, but when I worked there as a CPN, so 2019, they had a system similar but you had to get special authorisation to have access to the system at all, and only health professionals could. So if you were a care co but you were a social worker or potentially an OT, certainly the social workers couldn't access the system. ***P11 Nurse***    So, any prescribing that we do, we notify GPs in writing, which is just electronically sent to them. So, they get a notification from us when we’re prescribing anything in that sense. We don’t actually have access to the GP record, so we have to get, kind of like, a care summary from them to look at history prior to our, sort of, medical reviews. So, we communicate with GPs via letter or email and they tend to do the same with us. With mental health teams, it’s incredibly variable and I would say that’s based a lot on, if teams know us and already work with us and there’s established relationships. So, I think that is the key factor really. Often, we don’t get clear information as to which community team they’re involved in if they are involved in a community team. We’ve established some closer working relationships with some teams, I suppose, more than others, in that sense. And where we’ve got closer working relationships, it tends to be a pick up the phone or we certainly copy them into our letters if we’re aware that they’re involved with us. ***P25 Nurse***  Absolutely lack of communication. Because on that instant we had to go digging around finding what he’d been discharged on. It wasn’t automatically forthcoming. The care coordinator was having to kind of like ask for the information, because it’s obviously not on our system, automatically not on our system, so the care coordinator was having to ask. And I think even at the time of review we still weren’t absolutely sure exactly what he was… No, we were sure what he’d been discharged with. We weren’t sure how much clonazepam he was using when he was in. So yeah, it was just communication, yeah, absolutely. ***P31 ACP***    I don’t think it would be unfair of me to say if we had integrated systems that could share information more seamlessly that would go a long way in terms of people having access to contemporaneous information and negating the need to rely on someone actioning something to be sent somewhere. We have moved on an awful lot in that we now use electronic sending of documents. It’s not that long ago that we moved to that and we were still sending discharge letters to GPs in the post. I’m so disappointed to say it’s not that long since we stopped using our fax machine as well, so we’d like to do nothing more than light that on fire. I’ll not get into that. But I think that would go a long way for lots of different reasons. ***P32 Pharmacist***  Because you might leave messages they never get back to you or sometimes they're like, you're not involved with the patient and I'm like they are involved with me and this is what I've identified and it's just, I'm trying to tell you so you can either help me make a decision or so that you guys can follow up this patient in this particular way. And sometimes I just feel like it just falls on deaf ears, so sometimes I just write a letter to the GP, and I go, this is what I’ve found, I tried to contact this person, but I can't get any further. Because with the time limit, I've got of a week, it's just about that. And I suppose it's also, so it's the timescale but also, they're not my only patients. Sometimes I get pulled, honestly, I'm pulled in so many different directions you wouldn't believe. So I've only got a limited amount of time myself anyway. ***P37 Pharmacist***    This is the good thing about my role, is that I’m interface. So, I’ve had very many scenarios where I had to...if I had certain things that were, sort of, concerning or there’s some discrepancies or something like that. I just literally message the psychiatrist myself. And I know, ‘cause I’ve worked with these psychiatrists before, in terms of speaking to them over the phone for many years now. So, I just...luckily on Teams, I can just message them and then have a quick discussion. And I’ve got access to the mental health system, mental health patient records, so I can see what’s going on with that patient. And link in with the care coordinator or the medical secretary and it’s just so handy and I will just...for me, once I see the mental health records, I’ll be able to see, right, okay there is a plan here but the GPs not aware of this plan so I will just feedback to the GP that this is what’s going on. […] And it just helps smooth line that communication gap of what’s going on.  ***P7 Pharmacist*** | |
| A3. Discharge plans and medication reviews | What are the processes and plans set out, by health professionals and organisations, to ensure medication safety. What plans for medication review are made, who completes this review and how useful is it? |
| When I was discharged I was under the early intervention team, so I was assigned a care coordinator, and I was on like a three year pathway. So at first I was like oh my god, three years seems like an awfully long time, but actually it took me that long to get better. But within that time I think I had like three different care coordinators, but they were constantly making sure I was okay with my medication. So when I actually got discharged from hospital we went into the first national lockdown a couple of weeks later, so that hugely impacted my recovery because I had planned to pick up my life from where it was and socialise and eating out and doing all the things we used to do, and then I found myself just stuck in our house, not being able to go anywhere. So I really found that difficult. So the early intervention team were quite keen in the sense of being there to give me PRN medication. There was a few occasions where I had diazepam because I was really really struggling. Then what happened was I’d have quarterly meetings with the consultant psychiatrist at EIT and he’d obviously review my medication. **P27 Lived Experience**  Yes. So sometimes, so I did a quality improvement project about this when I was still a GP trainee. And we looked specifically at anti-psychotic monitoring in our practice. And I would say that whilst some people were still having appropriate monitoring, due to other comorbidities, so they may have diabetes and a diagnosis of, say, schizophrenia, so they would inadvertently have had some appropriate monitoring but not all of it. There was a huge proportion, I can’t remember the exact figures now, that hadn't had any monitoring. We didn’t know whether they were still under the care of psychiatry, or some of them had been prescribed anti-psychotics so many years ago, we didn’t know what the reasoning was. We don’t know, you know, what the indication of the medication was. And they’ve never even seen a psychiatrist. So this is before GPs were a bit more cautious, perhaps or were told that it was beyond our skillset to prescribe, so they may have been placed on a mood stabiliser for depression and actually, that’s probably not appropriate now. And so they’ve never had monitoring. ***P10 GP***    So if someone’s been titrated on the ward with clozapine, you have weekly blood tests for about 18 weeks, then two weekly blood tests for another 18, 20 weeks, then you can go to a four weekly blood test if the clozapine patient monitoring service tells you that you can. Highly restricted. Wards often don’t give enough thought to the fact that on discharge also, in order to prescribe the drug, the prescriber has to be registered with the Clozaril patient monitoring service or the equivalent for another brand. The only pharmacists who can deal with it are also pharmacists who are registered with the CPMS (Clozaril Patient Monitoring Service). Once you’re up and running it’s relatively problem free. You go along for your blood test, you get agreed on the blood test there and then, they put it into a machine there and then, you get your clozapine for the next however long and off you go. In order to get into that system they need enough notice to put you in, if that makes sense. ***P11 Nurse***    Home treatment teams are different, [voices overlap 0:41:17]. So when the patient’s discharged, we ask to go to any pre discharge meeting but we’re not always invited. It depends on how much forward thinking there has been about the need for home treatment on discharge. If they’ve thought about it for a couple of weeks then we will go to the meeting because they’ll have told us about it. Sometimes we get told on the day of discharge a patient’s been discharged. Sometimes we get told after they’ve gone. After they’ve gone we can only technically sign up for 48 or 72 hour follow up, but a lot of the time patients need a supported discharge, the staff just don’t want to tell us that because they’ve already let the patient go. So when we get there, basically on our first visit, we’re just doing a general assessment of, actually, how they got into hospital, how hospital was, what are they like since they got home. Not everybody will ask about medication, but I would. I was a care co for 21 years and I’m an NMP, so I say what have you been discharged on, how much medication did you get, have you spoken to your GP yet, what notification you’ve got. ***P11 Nurse***    I think that medication reviews have come to be seen as something really quick that you can do, a quick consultation, they’re often done by phone which I find impossible because people will say, well I’m taking that little blue pill in the morning, I have no idea what that little blue pill is. So, when I have somebody sitting in with me, well I’ll ask them to bring in their medication, but they don’t always, there are some missing but actually if there are some missing, then we say, well is it because you…you could identify that they’re not taking everything and they might need a dosette box but I think medication reviews have become very transactional. So, I don’t think much information… ***P12 GP***    So now what I do is, because they get yearly checks, so yearly physical health checks, so anybody who’s under the CMHT in our trust has to have a yearly physical health check. And that happens, that does happen. But what I do is I always ask his GP to do one after six months as well. And that’s how his diabetes was picked up and he’s on two diabetes tablets now. And also there’s not the same… Like he’s ended up on medication for the side-effects of the medication without very much explanation, he’s just been expected to… He’s a big lad now, he’s put on an awful lot of weight, and nobody has talked him through, especially at the beginning, actually, especially right at the beginning after his first admission, about diet and walking and all of that. ***P15 Carer***    R: And I don’t think people understand the worries we have as parents. The last thing I ever wanted was for my son to be full of chemicals. I’ve had to accept that without them he gets poorly. I don’t believe he needs all of them. And I think sometimes there’s a reluctance to properly sit… I’d love for somebody to sit down… Ten years he’s been in, in services, on medication. Surely he deserves for some skilled pharmacist to sit down and properly look at what he’s on.  I: Go through and look at everything.  R: Look at everything, and look at his body and his social life and his self-confidence, and look at everything. Because I think they’d change things, I’m sure they would, if they had the time to actually look at everything, but nobody has the time and they just go with what’s been done before. ***P15 Carer***    R: I think first of all, it’s a long time coming, having a mental health practitioner, it should have happened years ago.  I: Yeah, based in the surgery.  R: Because obviously now GPs are under more pressure. Even with the GPs a long time ago, it should have been put in practice, seeing a patient after discharge every three months. Checking to make sure that they’re taking their meds, leading a healthy lifestyle. You know what I mean? That should have been years ago. But I think now with these mental health practitioners, they need to see you. It’s no good just on the telephone. She never asked one question about my medication. Behind the scenes, I might not even be taking it ***P16 Lived Experience***    R: So, discharge…well, basically once…discharge was potentially planned, so people would have a potential discharge date, it doesn't mean to say that they would get discharged on that day. But when someone comes in, you're automatically working towards a discharge, making sure that there's things in place to support the patient really. Medication is either blister packed or put in dosette boxes and then handed over to either the patient or the carer on the date and time of discharge, along with a discharge summary goes to the GP.  I: And is that regularly that they’d be in dosettes or blister packs, you wouldn't just get bags of boxes of medicines then, it would always be like that would it?  R: Well, it depends on how many medications the patient has, but usually handed in a sealed bag or the nurse would have to double-check their medications prior to discharge to check that they’re up to date. Because there has been…you can get discrepancies where if you've got a date of discharge and then obviously the patient, for whatever reason, doesn't get discharged, then that medication would then have to go back to pharmacy and then a new prescription be prescribed. ***P17 Nurse***    R: And it's not just mental health meds, it’s physical meds. They're not been called into a GP practice, they’ve not had a review, they've not had a physical health check. So, there should be someone in primary care that links in with patient, mental health patients especially and does these tolerance checks, does these follow-up calls, looks at the…you’ve got a community pharmacist, well who's checking that these patient…why are these patients not having their medication? They’re not having it for a reason because they've either got mental health problems with no support networks or they’ve got a memory problem, so they’ve not been getting the medication or they’re getting the medication ad-hoc because of no support networks or the family member thinks they’re taking their medications and fully capable of doing it, yet they’re sitting on a GP database and the medications have not been issued.  There needs to be…I know you've got your primary care, and I know you’ve got your mental health nurses in situ, I think there needs to be more onus on the mental health nurse on your case allocation, when did your patient last have a medication check? When did they last have it issued? ***P17 Nurse***  So we would see the discharge and any changes made to the medication. That would be looked at by the technician or the pharmacist and then any changes would be made to the medication. A conversation would then take place to discuss the changes. And then normally there’s some additional information, so there might be a need for a mental health review afterwards. So definitely it’s not protocol but it’s good practice to check in with the patient over the phone, or physically calling into the practice and just have eyes on them, make sure what the state of their mental health is at the moment and follow up with them as well. So that’s normally what would happen. Any further referrals or recommendations from the discharge, then we will put that in place. So any additional counselling they request us to do or any referrals to other teams like drugs and alcohol, we would then look at that. Technically, it is referrals they should be doing but sometimes we do see it where they will say refer on. ***P18 Pharmacist***    Yeah. So I think around definitely reviews, reviews are really important [voices overlap 0:08:41]. Reviewing compliance. Are they actually taking the medication? They’re not going to feel better if they’re not taking the medication. It’s going to be no use. Sometimes just encouraging them to take their medication. Finding out why. So more often than not we make our own assumptions why they’re not taking medication. They might not be able to take them. So finding that out in a review is really helpful to get fullness of… I see it often what the younger patients just I’m so busy, I forget to take it, and then finding ways for them to help them remember to take the medication. There’s so many apps now, so they like a good app, medication reminders. ***P18 Pharmacist***    Okay. I think sometimes the understanding. So maybe it’s not clear what they should be doing with their medication. That can be a barrier. Say they’ve changed their medication, it’s not been made quite so clear on the discharge. Nobody’s spoken to them. I think that’s why it’s so important after discharge to actually speak to the patient. Some medications that…maybe a cream you might not need to ring the patient, but patients in terms of mental health, they must be rung every time they come out of hospital and prioritise that communication with the healthcare professional. I remember when DMS came out, discharge medicines service. When that came out so anyone that had been discharged from medicine it would go to the community pharmacy as well because previously we never saw the discharges. So changes that were made in hospital we’d never see. So that was quite influential and helpful in actually community would be aware of the changes that were made in secondary care to the patient’s medication. ***P18 Pharmacist***    Well, not a formal review. When he has seen the psychiatrist, the psychiatrist does discuss, do you think this has helped you, do you think more of that’d help you. So, that’s a review in a way, but not as… [Inaudible 02.58] the GP, you would have a pharmacist phone you. This is for physical health [voices overlap 03.03] and who did an independent review of your medication […] So, you don’t have that with mental health. ***P22 Carer***    They do. They go through each tablet. I've not had a pharmacy review for about a year. But they do go through every tablet. And they ask me if I know what each one is for. And basically, I know all the basics of them. If there's anything I'm unsure about, or I feel I've had any side effects, I will ask, I will tell them. And it's the same when I have the review with the nurse. We go through me conditions and things are explained. ***P23 Lived Experience***    Yeah, so with the lithium, I have to have my bloods done every three months, and then because of the eating disorder, I have to have an ECG every six weeks. And my ECG was slightly abnormal, which everyone put down to my eating, and actually the GP reviewed it. He did my lithium levels, and he said my lithium levels were too high, so he actually reduced the dose of the lithium slightly, which made my ECG go back to normal. So, that was helpful. He’s been really, really supportive. ***P26 Lived Experience***    I suppose the only thing would be, say, if I did want to reduce…and it was quite useful, because obviously I was only on aripiprazole for, I think it was six months, and obviously I didn’t have another relapse or anything, so I was obviously weaned off that. So I think in terms of that process and having the GP and the early intervention team who helped me with that, that was useful. And then also I suppose another thing, while I was actually on this three year pathway, so I’ve got my eldest daughter and then while I was under the care of the EIT I was pregnant with my second daughter, so there was a period of time where through my own personal choice I decided I didn’t want to be on any medication. So I was obviously advised about the fact they believed it was relatively safe, but [voices overlap 0:12:03] it just felt like it would exacerbate my current mental health, worrying about if [voices overlap 0:12:11] happen. ***P27 Lived Experience***  Yeah, so I’ve never had a medication review with my GP which I think is, sort of like, well a doctor rang me the other day for a study, and he asked me what medications I was on. And I told him, and because my BMI is really high and I’m diabetic, and I said to him that I’m only on metformin for it, he was like very, very shocked and concerned, and said that I should be on some of these, like Ozempic and other injectable medications, and that I should speak to my GP about it. And basically, in summary, it’s kind of like I have been worried that I’ve kind of slipped through the cracks a little bit, and just been given whatever specialists have said, ‘cause I’ve been under different specialists for different things. ***P28 Lived Experience***    And then in terms of healthcare professionals and teams and stuff, I think, yeah, the GP surgery I'm with is really good. They do annual health reviews. So they do things like all my…they do the MOT kind of thing every year, and then that includes a lot of discussion around the medication I'm on and doing bloods and stuff. ***P29 Lived Experience***    I think what the team don’t take into consideration is doing a full medical…medicine review, sit down with the patient and go through some of the questions a patient may have, for example, these are the common side effects from the medication, so I've been taking the tablets for this certain amount of time, you know, and this is what I’ve noticed. But under the team that I've been on and the treatment, I’ve never come across where the clinicians have actually listened or, you know, taken my views into account. So I think it would be very helpful, I have to say, like, if there was a better medicine review because…to support one’s…patients’ recovery and wellbeing, it’s very appropriate for them to be on the right type of medication…***P30 Lived Experience***  So that was that side of things, but quite often we are one pharmacy team, so the inpatient ward pharmacy team work directly with me anyway. We’re part of the same kind of department. So if they think that there’s someone that needs specific follow-up or there’s specific information that I need communicated to me, they will flag the person when discharge plans are in place. So, again a good example of that was someone who whilst in hospital had been commenced on an antipsychotic depot injection and the reason that they wanted me to get involved was mainly from a sort of procedural perspective in the first instance, getting the prescription charge written, getting the prescription written to actually procure the medicines, and make sure that all of that side of things was set up. But also, they had been quite involved with the client during the admission because it was a new medicine, counselling them on what to expect and following up in terms of things like side effects and also therapeutic efficacy. I suppose that’s more traditionally what the medics and the nurses on the ward might do, but we’re definitely involved with that as well. So they had suggested they had been quite involved and it might be helpful if I were to continue that line of work after this person was discharged as well. ***P32 Pharmacist***    Okay, so what happens is usually if they are well and they are stable and the mental health team are happy and then we are continuing the prescribing, we basically add it to their repeat prescriptions. That will either be done by a GP or one of our pharmacy team and then we will put a medication review date in their records and that could be three months, six months or a year. So, if they are well, they are stable, there is no concerns, they are not coming off the drug any time soon, genuinely I might put a twelve-month review. If it says the mental health team will follow up but we’re continuing until we are told otherwise, I’ll put a twelve month review in place and then what will happen is, when that review date comes up, assuming the patient doesn’t contact us sooner saying there is an issue, when that review date comes up, either a GP or one of the pharmacist team will have a telephone call or a face-to-face appointment with them to review it. But for some patients, we wouldn’t contact them for a review, we would simply change the review date and do a review from their records. So, for example if they are on a depot injection that’s been issued by the hospital and they’ve sent us up-to-date letters and the patient is engaging with the service, we would probably just automatically change that for twelve months because there is no real action there for the GP. Then usually during those reviews, we ask things like, do you know why you are taking it, do you know how to get it from the pharmacist, do you have any issues getting it out the container. Are you using anything over the counter on top of that or any drugs on top of that. ***P35 GP***    No. I never felt like I had any real clear pathway that was going to be followed. I was very confused the whole time. I felt lied to constantly because even if they weren't intending to deceive me, I was just constantly told one thing and then something else would happen. It was…there was never a clear expectation. There was never a clear pathway laid out to me. I was told every day for two and a half weeks that the psychiatry team would come to see me that day, and it wasn't for two and a half weeks that they did. I was self-harming, I had cuts everywhere and the nurses weren't doing anything.  ***P5 Lived Experience***    So, I think making people a) understand the benefits of medication and why they're taking what they're taking? Because so many people I know have just been put on things that they have no idea what they are, no idea why they're actually taking it. Also making a tangible goal for dosage range. A lot of the time it can be really helpful to have a target idea of what your goal is. That was again something my psych did, he was like, we have this range, we can take you to the lower end or the higher end if you need, but this is the range we're looking at. So, giving I think a tangible goal can really help and giving that sense of a plan. I think having regular contact in regards to your medication, particularly when you're first taking it is very, very important. Even if it's not the psychiatrist checking on you, I really think having a call a couple times a week for the first month/two months taking it of someone you trust at a service would be genuinely really helpful. ***P5 Lived Experience***    Clinician and primary care. So, what usually happens...now it depends on which pathway that they’ve come. So, if we’re looking at inpatients, the patients are discharged with a discharge prescription. Usually given one or two weeks. And it depends on whether there is a risk assessment being done, some patients are given a week, some are given two weeks. It depends where they’re going forward, obviously, ongoing forward management plan. Now, when they come...now, depending on that pathway, say, if they’re discharged to the GP. Then electronically the discharge paperwork will go to GP system. ***P7 Pharmacist***    So, what will happen is, somebody will be discharged and if they’ve got no ongoing care, then they tend to be stuck on what they’re discharged on because I think many GPs feel, I don’t want to alter that dose of quetiapine or that dose of lamotrigine, it’s not within my competency. I mean I will sometimes tinker with people’s meds, so I’ve got a patient at the moment who was started on quetiapine a few years ago by a psychiatrist. She’s not got any follow-up now; she’s trying to get back into work but the quetiapine makes her tired. So, we’ve reduced it a bit, just to see if that can improve things but actually that’s made her more anxious. In fact, with this particular person, I’ve actually referred her back to CMHT saying, I need some help here because she’s really unwell, she’s certainly on quetiapine, lamotrigine and antidepressant, I can’t add anything to that, that’s not with my competence. ***P12 GP***  The frequency with which the consultant at home treatment has to emergency medication reviews for patients post discharge is farcical. It should never be needed, because that patient has just come out of an inpatient stay, why in God’s name within a [week or two do they need to see a community consultant to review their medication, they absolutely shouldn’t. They’ve just come out of hospital. What are you releasing them for? Again, that’s because the patient either can’t continue on that medication for whatever reason, whether it’s habit forming or again it’s got side effects that they just can’t manage. ***P11 Nurse*** | |

**Theme B – Fragmentation and lack of continuity of care with medicines for patients and carers**

| **Sub code/Theme** | **Narrative description** |
| --- | --- |
| B1. Lack of continuity of care and diversion of responsibility | Responsibility is unclear or is diverted and passed between healthcare providers and staff. There is a lack of continuity in care and medicines because the collaborative networks are fragmented. This impacted upon patients. |
| Well, I think to have somebody in the community, a CPN, which [name] had at the time but doesn’t have now, gives that continuity. Because certainly in (NHS Trust name), which is the authority we’re in, it is all very hit and miss. I mean, the psychiatrist who deals with you in the community may not even know what medication you’ve been prescribed. So, it’s all very hit and miss. It’s not good. ***P22 Carer***  There’s a lot of silo working sometimes. You know, working in silos, in that the GPs, we will have our guidelines about what’s appropriate and we’ll have our idea about what is safe and what is not safe, I guess, and the risks, you know, if we’ve ever got any doubt that this person is not going to keep themselves safe, then they should be, you know, reassessed, whether that means readmission, that’s not our decision. I’ve never been involved with a sectioning or anything like that, so that would be a separate interest, I guess. ***P10 GP***  I think in primary care we're all very hesitant, all of us, very hesitant to make changes, especially when someone's just come out of an acute or long-term mental health admission. Because the assumption when they're being discharged back out is that they were stabilised and everything was fine. They're good on what they're currently on, what you've left them on. And so nobody really wants to make those changes or rock that boat, because we really don't want to make things worse. Because the risk is there, the risk that they may get readmitted is there. And that's more acute when they've just been discharged. ***P33 Pharmacist***  No, absolutely not and so, we then have to…then you are in a difficult position because you are either telling the patient to contact their mental health team back or you then have to contact them to find out what’s going on and there may end up being quite a bit of toing and froing and the patient is stuck in the middle of it, basically. If it’s something that we’re very comfortable with prescribing like an antidepressant, like an SSRI, it makes things a little easier because if they’re not an overdose risk, we can go on a risk verses benefit and go, while we are sorting it out, I’ll give you a bridging prescription. But when it’s an antipsychotic, it’s…so there is some stuff that is a flat-out red, we do not prescribe, we are not allowed to issue at all, like depots, but the antipsychotics fall in this horrifically grey period where guidelines are very much, GPs are not to initiate until stable. But then some of the GPs in our practices have done psychiatry placements or come from a psychiatry background. So, they feel very competent clinically to maybe do a bridging prescription for the patient’s best interest. But then there is some of us who, and I include myself in that, who are more newly qualified who don’t have that experience and we can’t. So, actually it ends up being which inbox of the GP you end up in to which type of care you get. So, it’s not consistent. ***P35 GP***    R: So, I’m going to be honest, I don’t know because no-one tells me as a GP, you have to understand that I literally get this letter saying FYI, we’ve done this, more information to follow and then I might get a letter at some point down the line that tells me the context to that decision being made. But we receive so many documents in a day at the GP surgery, that might not actually come to me, it might go to one of my colleagues. So, it’s not until a problem happens sometimes that I then have to go through the records to get some understanding to where a decision was made. But I’m going to be honest, the standard is never to explain why a decision is made, we’re just simply told a decision is made, unless there was a drug side-effect and they’ve specifically said, this drug was started, this drug was changed because they had this drug side-effect. ***P35 GP***  It’s just a theme running through my thoughts about secondary care mental health is that it’s very unclear who is responsible for what and it seems it’s like a game of tennis. You’ve got a patient you’re worried about and you bat the ball out to someone who you think is going to help. They bat it back and say, no, not our problem, and then you spend ages trying all these different people to fix… What actually needs to happen is there needs to be a system where GPs can refer somebody on that they’re worried about and then if that person thinks it’s not their responsibility, it’s up to them to then pass it on to whoever’s responsibility they think it is.  I: Yes, rather than passing back.  R: So then doctors won’t have to play this ridiculous game of tennis, which is a total waste of our time, because we don’t… There are other things we could be doing that would be a much better use of our time than playing tennis with mental health services, I think. Why can’t you just refer to the simple service that will take responsibility for it and sort out whoever needs to see them? ***P9 GP***  Yeah. Side effects have to be manageable, that’s the other thing. [Voices overlap 0:35:44] doctors don’t often really care about the side effects, what they care about is removing the acute phase of the illness, quality of life and ability to manage that quality in the community. It’s not something that most of them are overly fussed about because most ward staff and ward doctors have never worked anywhere else. They don’t really think about that. Whereas as a CPN that’s all I think about is actually can that medication regime be managed and maintained in a community or can’t it. ***P11 Nurse***    I think what happens is, if they’re not under secondary care and they don’t have a key worker or a CPN, then they get stepped down, they call it, rather than discharged, back to the GP. That causes a lot of concern because…well, what I hear is that the GPs don’t understand a lot of the psychiatric medicines, that’s what I hear. And that they’re also a bit reluctant to change anything. So you can go on and on and on for ages with no changes because they’re reluctant to change anything. So you wouldn’t know if your loved one or yourself could be better or on anything different because it’s just not… Like they’re just going with what the psychiatrist has said when you were discharged. ***P15 Carer***  And obviously, like everywhere, it does worry me, that’s something that has concerned me since being out of hospital is I think because you’re seeing so many different doctors now, you’re not seeing anyone who’s like a regular face, and then it’s also that concern of yes, they’ve got notes but I think it’s having to repeat your history. Obviously it’s not, because we don’t live in an ideal world, but in an ideal world it would be really good to have someone who knew what had happened to me. Not necessarily to go into great detail but just have that basic summary about my previous history. ***P27 Lived Experience***    I think sometimes there are gaps in the spaces between services, if you see what I mean. Like the GP can be the best GP in the world, but if they're not hearing from the community team in time or they're not receiving the discharge summary in time then they're quite limited in what they can do or what information they can give you. So I think communication is really important. Not just with patients but also between the services that are linked up. ***P29 Lived Experience***  So as soon as we start the discharge planning process it’s normally the, I hate using this term, but the junior doctors on the ward who will start populating that information with the events leading to admission, et cetera. And now we have an electronic prescribing system in place as well, so that makes it slightly easier in that it pulls that information digitally rather than relying on someone entering that in free text, so that reflects all of the information of the medicines that someone was prescribed whilst they were in hospital. But again, that gives rise to some difficulties as well because some people might have as and when required medicines that are mainly for hospital use and it might be that they’ve remained prescribed but they might not have been used for several days or weeks prior to discharge, but if that’s communicated on the discharge summary the colleagues in primary care who are receiving that information don’t necessarily have that context, they just know that it’s prescribed there. ***P32 Pharmacist***    I suppose it all starts with are they taking their medicines? So I think we start from…as a pharmacist, we start in steps, which would be do they have what they need? Do they know what they need? Are they taking what they need? And actually then, there's that whole grey area of is this somebody who's come straight from an acute inpatient stay, stayed in mental health for quite a while? We've had clients that have been in mental health for 18 months, for example, and they've been treated all the way through and then they come back home or into community, into primary care, and then all of a sudden this is a shared care agreement. And that sound reason, geography and location, and counties, that may different for that. So I can understand that that's a complete different level of discord there that doesn't necessarily get communicated across. Because six months down the line, how's anyone supposed to remember that that got started there in that…whilst the patient's in a hospital. And it can't be quite so easily continued because we're not sure what the continuation or the monitoring process is for that. I think in terms of the continuous monitoring and support that we offer, that may differ between different practices as well. Our team works across a small number of practices, there's only five or six practices that we work in. But they all work differently. So some may prompt an automatic booked telephone appointment with the patient post-discharge, and that's regardless, it's not because it's mental health. It's because they've come out of a hospital facility. ***P33 Pharmacist***    Yeah, like mental health teams, I suppose as well. So I know there's, so sometimes when patients get discharged, they go to local mental health teams, who are due to follow up patients. But even trying to speak to someone within those teams is really difficult, even when I know there's someone involved. Just because I suppose in terms of accesses and things, it's necessarily an easily contactable way. This is just me chasing as such because I might, I've got a few numbers saved over the years because of different times I've been involved. But often it's ringing one of these numbers and saying I need to speak to someone about this patient and then sometimes it's saying, I don't know, it just it always seems like a barrier. There's never anyone or people who are keen to hear my phone call. It's just such a big barrier. ***P37 Pharmacist***    That's the problem as well with the separation of care, I totally understand why the NHS has gone down the route of specialisation, because it allows you to have greater knowledge of your area, that's why GPs can't do everything, but the problem is for people who have complex care needs, it ends up being entirely disjointed and you don't have cohesive care. ***P5 Lived Experience***  Whereas, yes, so the decision-making from secondary care, we don’t always know when they’re due to see the patient next. We also don’t know whether the patient always attends. So we may get a letter to say they did not attend and then it’s a bit of a tricky time, whether they’ve actually been discharged or not. Because I would argue that anyone who’s known to secondary care, in terms of their mental health, under their care for medication or treatment, if they’re not engaging, that’s a sign that they need additional help, not that we should discharge them from the services. ***P10GP***  And the (place) based were absolutely useless. I was getting really, really bad, I wasn't sleeping, I was seeing things, I was hearing things. I was terrified. I was very ill. And they kept promising me that I would have a psychiatry appointment to get help with this, to try to get some kind of medication, some kind of help, and they literally…they told me they'd get me one and four times then they said they weren't able to. They told me it was going to happen and then withdrew it. Meanwhile I'm spiralling, I was having issues with drinking and coke. I was begging for help. Not to be…I don't mean this to be triggering, but I ended up cutting myself in front of them, because after the last time they told me that they couldn't help me, and all they could do was show up to my house for 20 minutes, and the only thing left that I could do was go to A&E. And if you have a diagnosis of BPD at A&E they don't tend to really do very much. So I had nothing. ***P5 Lived Experience***    No. I never felt like I had any real clear pathway that was going to be followed. I was very confused the whole time. I felt lied to constantly because even if they weren't intending to deceive me, I was just constantly told one thing and then something else would happen. It was…there was never a clear expectation. There was never a clear pathway laid out to me. I was told every day for two and a half weeks that the psychiatry team would come to see me that day, and it wasn't for two and a half weeks that they did. I was self-harming, I had cuts everywhere and the nurses weren't doing anything. ***P5 Lived Experience***    …it's ridiculous. And for me I feel like so much of the focus with people who have self-destructive behaviours, who deal with mood episodes, so much of the focus is on controlling the behaviours rather than actually giving alternative ways to fulfil that need. Because all of the behaviours are fulfilling some form of need and instead of trying to work out what need that is and alternative ways you can fulfil it, you just basically get punished for the behaviours. And it's like cutting a dandelion off at the head, even if you get rid of one particular bad behaviour because you've been punished into stopping it, just another one is going to sprout up because you haven't actually sorted the underlying cause. And I think such a problem is that they’re so much happier to hand you a pill, than they are to actually engage with you in a meaningful, therapeutic manner. ***P5 Lived Experience*** | |
| B2. Delays for patients and carers | Patients and carers experienced delays in the provision of medicines. There were challenges for patients around the timing and timeliness of prescriptions and dispensing of medicines |
| Anyway, (name of care coordinator), didn’t work at the weekend and on the Saturday, I was getting more manic, and I needed some stronger sleeping tablets, so I asked them for some stronger sleeping tablets. They didn’t work. (Name of care coordinator), came back to work on the Monday and she could not believe I hadn’t had my ECG over the weekend, so come Monday, I was even more manic. And also, there was no prescription for the quetiapine going up, so he didn’t write a prescription for me on the Friday before he finished work, so (name of care coordinator), couldn’t do anything. But I was left, you know what I mean. So that could have been prevented, on the Friday, if he’d have put it up, if I had it straightaway, I might not have needed hospital, I might have been treated at home, you know what I mean […] Because (name of care coordinator), came from (other city), and (name of care coordinator), said, I can’t believe what it’s like here, I have to jump through hoops to get help for you. They weren’t treating me as an individual. You know what I mean? I need very early intervention. Just for an example, it was September 2019, this is when the adoption agency said no to me, and it was the first home visit so they never even checked me out. And (Name of care coordinator), said I was low risk and they never even checked me out, but they didn’t have much knowledge about bipolar yet then ***P16 Lived Experience***  And I guess when a patient is often discharged, speaking from the community pharmacy side, there can sometimes be difficulties in the patient obtaining their first prescription, because documentation isn’t received from secondary care to primary care, medication changes don’t always correspond appropriately. Sometimes the patients don’t understand why they’re on certain medication, so changes are made and it’s almost like change being done to them, and maybe them not being involved. […] I guess that’s from the community side in a gist of it, and I think sometimes that can make it really difficult, as a community pharmacist then, when you have to sometimes mop that up, and do an additional check and make sure that you pick up some of the pieces, in terms of explaining, which can feel, sometimes, quite weird for a community pharmacist who doesn’t have a specialist interest or any specialist expertise within mental health. ***P14 Pharmacist***  At the time of treatment patients often say to us in supported discharge oh, I was having this medication every night to help me sleep and then they stopped it when I went home and now I can’t sleep. Well, what was it called? And I can’t remember, and there’s no information on the system for us to see to know what they got. But a couple of goes and you can you can usually get it right because it’s only going to be one of half a dozen drugs, and then we say well, if it’s promethazine, buy it over the counter, if it’s anything else, talk about it. But yeah, it’s… ***P11 Nurse***    So on his discharge he should have lithium and everything. He's sent home from hospital with a supply of lithium. Anyway, because of the change of GPs which has come as a result of being discharged, somewhere in all of this the GP ended up totally missing his lithium off his prescription after… He got it for…so he was discharged mid-January, and then he got it right up to the end of January. So that's like two weeks he probably got it whilst he was in this rehabilitation care home. Then when the care home requested more, from what I can gather, the GP wanted a blood test. […] Even though it was on the discharge documents, the GP said he needed a blood test. And then there's been lots of queries, because we have had an inquest into it. And there is some fault there. But somehow it's been missed. Like the GP…one GP said well, yeah, he's been on it long term, give him it. Another GP saying no, I want the blood test before I will give him it. Anyway, it went from him… Yeah, it was the end of January. It went from the end of January right up until 6th March with no lithium. And in that time, he was sectioned because he'd gone downhill. ***P13 Carer***    So I'm ringing them frantically trying to get this lithium for him, because I know he's going to go downhill. But I didn't find out until…it was late February when I actually found out, and the only reason I found out was because there was a note on the side in his room saying do not give diazepam. So this was a complete other medication, it's not on his prescriptions. I questioned that, and they were like oh, you shouldn't have seen that note anyway. This is the care staff at the care home. You shouldn't have seen that anyway. So I'm questioning it. And I said, well, can I see his notes? Will you give me his notes? They didn't want to give me it, but I fought and fought, got it. Looked on the MAR chart and saw lithium had been stopped. And I'm like well, why? So I'm frantically ringing around. ***P13 Carer***    Anyway, I told my care coordinator about it because she had difficulty getting in contact with them. They arranged a discharge meeting and never told her. Anyway, I told her about the problems on the ward and she made some enquiries and they have safeguarding issues. And when I left the ward, patients came forward, and then it got shut down. So that’s the only time really that I’ve been took off the quetiapine. And when I got discharged, I ended up as an outpatient. I was so desperate to get back on quetiapine but (name), my care coordinator, she said, (participant’s name) you can’t do it yourself, you’ve got to wait for the psychiatrist. I said, I can’t wait a week to see the psychiatrist, you don’t realise, I can’t function properly. ***P16 Lived Experience***    So what happened was, this particular chemist, which is next door to my doctor's surgery, not long after I’d lost my wife, I noticed, because I check, all my tablets are there, so when I was taking my amlodipine, no, what happened is, I took all my tablets out and I thought what's this one here? And I suddenly realised my amlodipine is not there and then it dawned on me that this is a different dose of amlodipine. So when I went back to the chemist, he said, yeah, it is. There was no apology, nothing like that, no apology. Then when my tablets were delivered, this is maybe two years ago, there was four boxes missing, there was four boxes missing, and I thought what's happening here, oh, it's the warehouses fault. Then, I think this was the middle of last year, my tablets had been delivered and everything was there. And the week after, there was a knock on my front door, and there was a bag of medicines. And there were about five boxes that I'm on, but I hadn't ordered them*.* ***P23 Lived Experience***    And she’s sat outside the hospital with her clothes and everything and a massive bag of medication, with new medication in that I’d never seen before, that I had to research. No timetables as to what was to be given to who and when. ***P24 Carer***    So when my clozapine bloods were amber, I was very much at the point of saying, I don’t want to be on clozapine any more, having to have two blood tests a week, it’s too much, and I was like, I want to come off it. And everyone was like, oh, you need to speak to your consultant, you need to speak to your consultant. So I tried to get a message to her, I heard nothing for weeks and weeks and weeks. I was talking to my care coordinator, and she was getting in contact and (name of Dr) was kind of saying, like, how it’s fine, and we’ll just wait and we’ll review it next year, and it wasn’t very soon, and trying to get in contact with the consultant, she was really difficult to, yeah…they kind of thought because I’m not in any danger or anything like that, it can just wait, and actually it would have been really helpful to speak to her. ***P26 Lived Experience***    Well, yeah, I mean, ‘cause I’m on weekly medications, so it can cause quite a problem if they’re sort of delayed, especially over the weekend, ‘cause I don’t have like a back-up, you know? So it’s kind of like, if they don’t give me the medication on the date that I’m asking for is, then I don’t have anything to keep me, until they give it to me, and of course, I do ring up the surgery if they make this mistake, and I say to them, I need it, what can you do? And the receptionists, all they can do is apologise, and of course, I know it’s not their fault, but it’s just, you know, it’s happened recently, I don’t know, yeah, it does ‘cause a problem, because it causes me a lot of stress and anxiety*.* ***P28 Lived Experience***    So there was a lot of stress around the communication with the GP and getting the prescription sorted and everything. And then what I think maybe were the side effects of the risperidone, I mean it could have all been placebo effects, just switching could've just helped just on a placebo level, but it was interesting how within a few days of starting it I became very anxious and then within a few days of stopping it that kind of resolved. So yeah, it just… But those were the two main concerns around safety, because I didn't feel like…when I was feeling very anxious I didn't feel like I would make a very safe driver, because I was anxious about driving. So that was a bit of a spiral. And then not wanting a break in treatment because I didn't want to have a relapse or I didn't want to… And those are quite risky anyway. Like if you have a psychosis you're kind of at the mercy of whatever seems to be what crosses your mind is important and then you go off on this strange journey of following what seems to be significant information. And that can be quite perilous because you can end up in strange and dangerous situations and things. So I was worried about that relapse and…But once I got the medications, I got into the swing of picking up the medications regularly it was very…and switching, it's been really good. ***P29 Lived Experience***    And I don't mean to beat up on the NHS or anything, because I think they do a really good job, especially with everything that's going on with the NHS at the moment. And all the pressures that they're under and stuff. But I think those pressures are having an effect on the communication. And yeah, just more…I think there are periods of time where patients…after they leave hospital they just don't necessarily know what's going on. If the home treatment team come around for example, and they say is everything alright with your medication or whatever, and I remember saying to them I've called the GP, the GP is still waiting for the discharge summary. And then the home treatment team said well, yeah, ring the wards, and get them to do that, because that's really important. So people within the different services I think are saying the same thing as well. They're saying…or they're recognising that there are communication shortfalls in place. And some services are having to tell patients to chase up other services, and that's interesting. Like oh, you should ring the ward and you should really chase that. And it's like oh okay. But then, I don't know, say you've got the patient with really severe depression that just doesn't want to get out of bed, they're not going to be feeling…chasing up wards. So I'm very lucky that I've got a support network, like my partner's the one doing all the ringing people and stuff. So she's… And prompting me and saying you need to get your discharge summary and… That really helps. But yeah, I think it's just a combination of factors that can all be lumped together under communication in some way. ***P29 Lived Experience***    Well, like I say, the biggest problems I find are when it’s coming from out of area. So an example I had was a lad that we’d suggested he comes in for an informal admission. It was our intention to get him on a long acting injection of aripiprazole because we knew aripiprazole worked and it was just more a regular [background noise 0:07:00] problem. So the idea was to get him in on a long acting injection of aripiprazole. He went out of area, they decided that they’d put him on I think flupentixol. I don’t know how familiar you are, (name of researcher), with meds, but that’s an older style…[…] And it didn’t really suit him. He was really quite akathisic with it. So he’d come out on the flupentixol. He’d also been given clonazepam when he was in. So he’d come out with a week’s supply of clonazepam. He didn’t really know how to use the clonazepam, so he was all over the place, really anxious, really quite distressed. So I did manage to see him within I think about a week of his discharge. So reviewed him quite urgently, really tried to get to bottom of what his distress was about, and I think my primary concern was the clonazepam. It was that he’d taken what he’d been given over the weekend, he’d then gone without it, he didn’t really know what he was doing with it […] to be perfectly honest. Yeah. So my most pressing concern was really addressing the benzo withdrawal. So I also had concerns about akathisia because he was really quite distressed with that as well. He could barely sit still. He was getting up, having to walk about, having to stand up. I was concerned about that, but I wanted to address the benzos first of all. So we got him on diazepam with some kind of withdrawal plan and then he was reviewed again a week later, and then we looked at the actual long acting injection and changed that as well. So that’s a scenario where it just doesn’t work. ***P31 ACP***    So I suppose I'm aware of some of the urgent nature of some of the bits and for some patients, it's really important for their mental health and for them to feel safe and secure and things like that. I suppose just as a pharmacist, I know the importance of some of these drugs, such as Clozapine. I did have a patient who went home without some Clozapine one time, and I was like, this is like urgent, this needs chasing up now. So I look at it differently because I suppose I have an understanding and awareness of sometimes the urgency of these things, but patients often get quite upset when those bits aren't there, because I think it's just, moving, being transferred from an inpatient setting to a home setting can be difficult enough because there's lots of new challenges, lots of new things to address. And I suppose with my teams, because with discharged to assess we have input for a week after discharge, but we're very intense quite early on, because we kind of need to get things sorted. And I suppose we’re a new team and there might be a package care starting might be district nurses visiting and things and all of that, and I suppose just being at home again and trying to adjust again can all be quite overwhelming for patients. So sometimes just something as simple as not having your medicines there can just really upset patients and might tip them over the edge a bit, I find as well. ***P37 Pharmacist***    And obviously it's unfortunate because of the pressures on our healthcare system, but patients often just get chucked out for want of a better expression. So they don't always, and I suppose medication is always the last bit in that process. It's always it's like, medications here, off you go thing. It's not necessarily like, this is your medication, let's have a chat about this. This is what's new. This is what's stopped kind of thing. It's always one of those things that I think medication is always the last bit. It's always the last bit. It's one of those bits where, I just think that could, it should really be done in advance and it should really be done better where people are having a conversation either with the patient, or if the patient's not capable of having that conversation with someone involved with the patient so that they know. ***P37 Pharmacist***    So, he constantly…he was the person who I had to request my meds from and I could tell he was anti-medication. I had been…I told him a month in advance that I would need my medication filled in January this year. He forgot. I emailed about it and then they got mad for me giving them a short period of time. I was in a really, really bad psychotic episode at the end of last year and coming into January, it was the worst I've ever had. I was in like…wasn't just psychotic symptoms, I was in full-blown psychosis, and I needed them really badly. I didn't specify the exact medications that needed to be given to me, because they've been given to me every month for a year now, and he messed up my meds, he got me 25 milligrams of quetiapine rather than my usual dose. I was going home because I had COVID I needed the medication, and when I said, I don't know what I'm going to do, I’ve ordered my medication like a month ago. He was like, oh well, I'm sorry this isn’t the outcome that you wanted. As if I was like a drug seeker. ***P5 Lived Experience***  It’s very variable. I think, to be honest with you, again, it very much depends on how much the patient wants to engage with primary care. As well as how much they’ve engaged with secondary care. But, you know, as GPs, we’re very familiar with initiating antidepressants. And continuing them. So we have quite good guidelines about how appropriate it is to monitor, you know, for example, seeing someone with potentially high risk of suicidal ideation, you know, within the first two weeks of initiating an antidepressant, and then depending on how they’re getting on, it may be that they need another two week follow-up. Or you may then say, let’s leave it a couple more weeks but, you know, with strict safety-netting instructions to seek urgent medical care if, you know, their mood was to deteriorate or anything was to change or any thoughts of harming themselves, things like that ***P10 GP***    It’s the same problems with everybody, which is that people are only given a week’s medication, no matter what day they’re discharged. But the discharge notification rarely goes to the GP on the date of discharge. There’s no allowance made for if it’s a bank holiday, Christmas, anything like that. So maximum, if somebody’s discharged at nine o'clock in the morning on a Monday, maximum you’ve got, and you’ve actually got less than five days…  ***P11 Nurse***  Wards also, no matter how many times you tell them, don’t seem to have any conception or understanding of how community services work. For instance, if a patient needs a blister pack, Venalink, is the other thing they get called, there is a waiting list at most chemists for those and the waiting list could be months or years long. You don’t walk into a chemist and say I want a Venalink, and they go yeah, no problem. You do get the odd chemist that does but most people have a waiting list. So when a patient’s discharged from a ward they say oh, they could do with a Venalink, because I won’t remember. Well, you can say that, fine, but it’s going to be potentially months before that actually happens, what’s going to happen in the meantime. There’s no thought given to that. And if a patient is not going to be capable of taking the medication themselves and therefore will need support to go on and do that wards don’t tend to think of that either. ***P11 Nurse***  So, often it’s the patient who contacts us saying, I’m running out of medication, we don’t know what medication it is that they’re on and if they’re on atypical antipsychotics or lithium or lamotrigine, we would normally want a shared care agreement to start prescribing. So, the letter arrives, it sits waiting to go through our doc-man system and it depends on what the delay is but that can be a week. Then processes that the letter then goes to the prescribing team who have a look at it and if a shared care agreement is needed, they will highlight it to the prescribing GP. We have one GP in a practice who looks at shared care agreements, so there’s inbuilt delays in our practice as well as the delays that we experience in getting the letter in the first place. So, for the patient, what they experience is, oh my goodness, I’m running out of tablets, oh God the GP won’t prescribe, the hospital has discharged them and really, I think people find difficulty trying to get back in touch with the ward or if they’ve got a case manager or a care coordinator, then that helps because they can often negotiate it. But if they don’t have a case manager, they’re stuffed really. ***P12 GP***    Anyway, (name of care coordinator), didn’t work at the weekend and on the Saturday, I was getting more manic, and I needed some stronger sleeping tablets, so I asked them for some stronger sleeping tablets. They didn’t work. (Name of care coordinator), came back to work on the Monday and she could not believe I hadn’t had my ECG over the weekend, so come Monday, I was even more manic. And also, there was no prescription for the quetiapine going up, so he didn’t write a prescription for me on the Friday before he finished work, so (name of care coordinator), couldn’t do anything. But I was left, you know what I mean. So that could have been prevented, on the Friday, if he’d have put it up, if I had it straightaway, I might not have needed hospital, I might have been treated at home, you know what I mean. Because (name of care coordinator), came from (other city), and (name of care coordinator), said, I can’t believe what it’s like here, I have to jump through hoops to get help for you. They weren’t treating me as an individual. You know what I mean? I need very early intervention. Just for an example, it was September 2019, this is when the adoption agency said no to me, and it was the first home visit so they never even checked me out. And (Name of care coordinator), said I was low risk and they never even checked me out, but they didn’t have much knowledge about bipolar yet then. ***P16 Lived Experience***  So, we would wish to see the person face-to-face, which means we actually start that assessment process with the first contact. They will be booked for what we call a medical review, generally within ten days of discharge from the hospital. Can be slightly varied, obviously, if we haven’t got capacity but generally within ten days. And that would be either with a non-medical prescriber or a medic. To actually, kind of, just reassess where we’re at in terms of our prescribing and a plan going forward. Dependent upon risk, the individual would then be seen ongoingly, either weekly, fortnightly, or monthly by their key worker ***P25 Nurse***    Yeah, so with the lithium, I have to have my bloods done every three months, and then because of the eating disorder, I have to have an ECG every six weeks. And my ECG was slightly abnormal, which everyone put down to my eating, and actually the GP reviewed it. He did my lithium levels, and he said my lithium levels were too high, so he actually reduced the dose of the lithium slightly, which made my ECG go back to normal. So, that was helpful. He’s been really, really supportive. ***P26 Lived Experience***    So as soon as we start the discharge planning process it’s normally the, I hate using this term, but the junior doctors on the ward who will start populating that information with the events leading to admission, et cetera. And now we have an electronic prescribing system in place as well, so that makes it slightly easier in that it pulls that information digitally rather than relying on someone entering that in free text, so that reflects all of the information of the medicines that someone was prescribed whilst they were in hospital.  But again, that gives rise to some difficulties as well because some people might have as and when required medicines that are mainly for hospital use and it might be that they’ve remained prescribed but they might not have been used for several days or weeks prior to discharge, but if that’s communicated on the discharge summary the colleagues in primary care who are receiving that information don’t necessarily have that context, they just know that it’s prescribed there. ***P32 Pharmacist***    So I think being better at identifying what people’s needs are around that and having the right approach to asking about them as well. You don’t know what you don’t know, so the client won’t ask and you need to be the one to think through where the difficulties might be and specifically target them. So as part of your discharge planning do you have a regular pharmacy that you use, how do you normally order your prescriptions from the surgery, et cetera. Because we only really give a fortnight’s supply of medicines, because, again, it’s often about minimising risk and that’s not really changed over many years. I think in the past it was considered well, a fortnight is plenty of time for the discharge letter to get to the GP and for the surgery to update all the information on the system and for them to be able to turn around the prescriptions that people need to get their medicines. And in many cases that works, in many cases it doesn’t. ***P32 Pharmacist***  That's only actioned if it's put as a priority. If our in-house teams are processing that as a normal discharge, they don't necessarily put it to the top. And on a Monday, Tuesday, those are potentially a practice's busiest days, which means we could get…depending on how big your practice is, you can get hundreds of requests per day. So it's just lost in a 300 request pile. And unless somebody else has either contacted the surgery in a different way to say my name's in that 300 pile list, can you ping it to the top? Because we can. And it's too late. By the time you send us the letter and the letter says this is urgent, it's almost too late. Because it's urgent in a pile of 300. And we've still got to read that letter to see that it says urgent on it, rather than someone waving flags at us and being like yeah, please look at… I'm aware you might have hundreds… But I think maybe a lot of people don't realise that practices do get hundreds. It's not like we get 20. And we've worked in small practices where we may only get 30 letters a day, and that's nice. But I've also worked in practices where we do get 100 letters a day, and that's not even counting prescription requests. They can get 300 prescription requests on a Monday very easily, especially now that patients can request their own prescriptions over the weekend on an app. ***P33 Pharmacist***  Because you might leave messages they never get back to you or sometimes they're like, you're not involved with the patient and I'm like they are involved with me and this is what I've identified and it's just, I'm trying to tell you so you can either help me make a decision or so that you guys can follow up this patient in this particular way. And sometimes I just feel like it just falls on deaf ears, so sometimes I just write a letter to the GP, and I go, this is what I’ve found, I tried to contact this person, but I can't get any further. Because with the time limit, I've got of a week, it's just about that. And I suppose it's also, so it's the timescale but also, they're not my only patients. Sometimes I get pulled, honestly, I'm pulled in so many different directions you wouldn't believe. So I've only got a limited amount of time myself anyway. ***P37 Pharmacist***  I think just having more contact about the medication, even a mental health nurse could call you, I think that would be helpful and it means…there's a lot going…when you first go on medication, you can end up with loads of side effects at first, and so having someone to have regular contact with I think would make it a lot easier for people. Because the problem is a lot of the time what happens is, a psychiatrist will prescribe you a new medication and then they're like, okay see you in two months and you have no interim care or contact between then. So, there needs to be greater opportunities for feedback and closer monitoring of dosage. Also, I've been quite appalled at how lax psychiatrists have been with blood tests in terms of prescribing things and not doing routine checks. ***P5 Lived Experience*** | |

**Theme C - Patient and carer voice: Shared decisions, information and empowerment**

| **Sub code/Theme** | **Narrative description** |
| --- | --- |
| C1. Shared decisions | Patients and carers were involved in decision making in variable ways .Sometimes there was choice and shared decision making with patients or carers, at other times they were not involved in decisions. |
| But usually it’s the nurses, wellbeing practitioners and then if the patient has a care coordinator, you know, they’ve got a CPA (Care Programme Approach) and they’re in the CMHT. Depending on the risk assessment there will be regular, kind of, follow-ups with the care coordinator. So, the care coordinator will, you know, as part of shared decision making with the patient, do a telephone appointment that...you know, regular catchup, check-ins, how are you doing with your medication, do you have any side effects. So, they will do that kind of screening or if telephone is not appropriate, they will actually go to the patient’s house and see how things are going in terms of their medication. ***P7 Pharmacist***  I don’t know, you would hope that they are involved, we talk about shared decision-making, so you would hope that the plan has been discussed with the service user before discharge. I think it’s very variable and it also depends on the health literacy of the patient and their capacity at the time. How depressed they are and there is always the odd person who goes, oh I don’t want to know, you’re the doctor. But I think people do talk about feeling that they’ve been discharged on loads of meds and they aren’t sure what the plan is either. In the short-term, they don’t know who is going to prescribe it and in the long-term, am I staying on these forever. ***P12 GP***    I’ve literally just had a conversation with my colleague earlier about this, about, yeah, we have to do the coproduction side. Patients have to be involved in that choice, and that Choice and Medication website, really supports that, and the literature. So, showing someone the factsheet, your doctor said that you have depression and we’re going to start you on an antidepressant. These are your options, this is what the side effects are, this is what it could do, this is how it potentially works, these are the consequences of stopping. What do you think, what’s…? Because we actually may make a decision, and it will be biased, based on what might be important to us, but actually might be very different to somebody else. So, I remember actually counselling a patient on a certain, like what antidepressant would you like, these are your options? And he was a (specific detail) athlete, a footballer, and I remember saying to him, I remember assuming in my head that I didn’t think that he would want mirtazapine because it would cause him to gain weight, and obviously because of his job…actually that wasn’t important to him. He chose one that was going to be less likely to cause sexual dysfunction, that was important to him, because he had a relationship. ***P14 Pharmacist***    Well, it's a joined approach, isn’t it, so it's teamwork, it's partnership working, it’s putting the patient at the centre of the care, the patient’s fully involved in the care. They know why they’re having their medication. They know the reason for the medication. They’ve provided consent to the medication. They’ve been given information about their medication. They’re aware of the avenues of support, your GP, social services, the memory service. And they’re given contact numbers as well, key contact cards. So, they're aware of who to contact and when. ***P17 Nurse***    …we’re all different, we’re all individual, so it’s a sort of person-centred approach really, one size will never fit all. And actually with some people, if they think that they are being supervised, overly supervised as regards their meds, that actually can get their back up and it’s not conducive to positive recovery. So I think the key thing really, and it is important to have regard to what family and friend carers have to say because they’ve got some very valuable lived experience insight into the person they care for and they are experts in their own right in that regard. And it’s good to get the patient on board, understanding why it’s important to involve their carers, because again when someone’s very unwell, they can become suspicious of the very people who are doing their utmost to keep them safe, you know, don’t share information with them, et cetera, which can make it very challenging for carers. So sit down with the family and friend carers, but don’t do it secretly behind the patient’s back because it can give this conspiracy complex. Make them aware of why it’s important, and if at all possible, have the conversation in their presence so they’re not wondering what’s been said when they’re not there. But it is key as well, of course, to sit down with the patient themselves and talk to them about what would help them with their meds following discharge and make a person-centred discharge plan on that basis, co-design it, co-produce it. Because when someone’s involved in the planning, they feel a valued, respected part of the team and that is a good foundation, isn’t it, then for going forward positively ***P2 Carer***    I think it’s that plus always putting the patient at the centre really. And actually, trying to recognise what the challenges are for each individual, you know, so that actually you develop a more person-centred approach to medication management. Some people don’t need any handholding and manage it very well, other people need a great deal of support to understand and get the best from their medicines really. ***P25 Nurse***    I: In terms of decisions around your medicines, how involved have you been in those decisions?  R: I feel like I have been involved. I mean, obviously, some of the meds I started when I was really, really poorly and didn’t necessarily have capacity to either agree to them, or know what was the right thing, and that kind of thing. I mean, it got to the point I was just like, I’ll try anything, I just want to feel better. I had been told I had to come off the clozapine the first time, because my physical health was really […] difficult, because it was the only thing that felt like it had helped, and then I was like, I’m losing it now, it’s not fair. So I kind of, when the doctor, ‘cause I got a new consultant, there was a new consultant started, and she was like, well we’ll just give it another try. ***P26 Lived Experience***    I ended up finding an obscure case study of a group of patients on risperidone who'd had similar…who were part of a similar subgroup. They'd had paranoid psychosis and they'd had ideas of reference. And it was specifically these patients that reported anxiety as a side effect of risperidone. So I said to the GP like I find this really obscure paper. I'm not saying it like definitely confirms anything, but I think that's really interesting that in some people it (risperidone) can be related to anxiety. And it's one of the listed side effects. So then after a while the GP said okay, would you like to speak to our mental health specialist? And it was her suggestion to look at switching. And when she suggested it I was very relieved. I was like oh yeah, okay, brilliant, because that's what I really wanted to do. But I didn't feel that the GP or the community team were particularly keen on the idea. ***P29 Lived Experience***    If somebody was blatantly experiencing psychotic symptoms that I thought could be better managed through antipsychotics, I always thought I had to go there. But now there’s certain clients who I know their attitude is already I don’t want it, and it gets them angry when you discuss it. So I have started to accept that I’ll tentatively feel around the subject and I’ll kind of see if there’s any change in their attitude towards it, and if that’s not apparent then I do feel comfortable, not necessarily overtly bringing the subject up. I might get criticised for that at some point, but I kind of feel justified in doing that in terms of okay, I could go the opposite way and just satisfy risk, satisfy that it’s documented that I’ve told you that you need medication. But where’s the benefit in them walking out and never coming back and seeing me? ***P31 ACP***    I’ve seen enough cases in the past where we’ve gone down a route collectively of putting someone on treatment, and I use that word putting deliberately, and because there’s been little engagement or that shared approach to making those decisions, people just stop taking it and they don’t tell you. So that’s what I always say to people as well when we are making decisions about it, if you want to stop taking it I understand that might be something that you want, just please have a conversation with me before you do that so that I can understand what the problem is, see if I can help find a way around it and we can manage that situation accordingly so that we’re avoiding anything like withdrawal effects. ***P32 Pharmacist***    So if you want to go off and troll the internet and go on google that’s fine, but bring it to me so that we can discuss it and I can put things in context or I can explain what it means. […] I think as well it’s almost an implicit way of sharing that decision making again because I’m encouraging them or hopefully empowering them to try and take ownership of this is for all intents and purposes your decision to make, I’m just here to give you the information that is relevant to you in your specific context, and I can make a recommendation but I’m not going to tell you either way to take this or to not take this. So that’s still a bit of a bewildering concept for some people, but again taking the time to build that rapport and engage with people helps a lot and goes a long way. ***P32 Pharmacist*** | |
| C2. Provision of information to patients and carers | The ways in which patients and carers were given information about their medicines. |
| Now, within that, there are easy read leaflets, there are things, literally with pictures on. So this is quetiapine, this is how you pronounce it, this is what it might make you do. And it’s about pitching that to the right level for someone to be able to understand. So it’s useful for children, for people with learning disability in terms of pictures, and then also it’s got more detail in there for those that want more information. Because, let’s be honest, giving someone a Patient Information Leaflet, you might as well be giving them something in a completely different language, ‘cause even we don’t understand what half of it means. And if you want someone to not take their medication, you absolutely make sure you put that Patient Information Leaflet in that box, because if you read those side effects you’re never going to take any of them. ***P14 Pharmacist***  So if you ask them particularly about medication, then they’ll explain it to you. Whenever he’s been on… He was tried on Clozaril, we had an awful lot of information about that, but he got red flags, his body went poorly, his blood, so he was taken off of that. But there’s always lots of information about the depot, you do get a lot of, this is an antipsychotic, he has to have it every month and it’s very important, and all that. But hardly anything about the orals, you’ll just get told, well, this is just another antipsychotic. I wouldn’t know whether they interact or whether they… They don’t really say a lot about side-effects until you’ve got them and you bring it up, and then they’ll say, oh, that’s a side-effect of… That happens in the community a lot. ***P15 Carer***    Sometimes what happens is when they come into the community either the colour of the tablet changes or the shape of the tablet changes, then they will say, it's not the same one so I’m not going to take. So, you have to explain. Or sometimes we got a tablet one milligram looks white, but half dose is blue, so some people want only blue. So, when you give the white one, they won't take, so you have to be a bit flexible and say okay, we'll give you two blue ones. So, they will take. Then they will say, I don't want two blue ones, I want only one blue one because in the ward I am taking only one. So, you have to be very flexible, you can't say like, oh no, this is what you are going to take. So, we have to work with them, we have to engage with them and sometimes once they come into the community if they develop any swallowing difficulties, then you have to change either to liquid form or like an oral disposable. Then again, it changes and again you have to explain, okay, this is what we are doing, it may look a bit different, the colour may be different. So, we have to prepare them in advance […] Without any prior discussion if you change them suddenly, they won't be happy, and we can feel in their position we’ll feel the same, like you can’t just take control of my life without discussing anything with me. ***P19 Psychiatrist***    And the thing about when you’re in hospital, you’re not generally given – in my experience – the patient information leaflet from the medications, and so often a patient will only actually get to see that when they’re discharged home and their prescriptions are coming from the local pharmacy. And not everyone reads the leaflet, I have to say, but in my experience with the people I’ve been a carer for, the two ladies, and they’re very intelligent and they have read them and they thought, I don’t think I should be taking this. But then they’re a little bit [inaudible 09:21] about actually raising that issue with anyone, because when you’ve been very unwell, you’ve often lost your confidence and you’re thinking, these…the clinicians know best, they must have already thought about that. ***P2 Carer***  It’s helping people understand, you know, their condition, their diagnosis, if you like you know, we’re part of that process of helping people make sense sometimes of what can just feel like a label that somebody’s given, you know. So, I think it’s that bit that you don’t just literally work with the drug use, you know, you work with the whole person. And therefore, that wider information is really important and helps us look at our own, kind of, risk management and look at where their substance use interacts with other things that they’re taking or doing. ***P25 Nurse***    Yeah. I mean, like a prime example would be, like a lot of antipsychotics have potential side effects as weight gain, which as someone with an eating disorder is terrifying. […] And it really puts you off, and actually that medication might be the one that really, really helps. But I think what would be really helpful, would be, instead of getting a list of side effects, maybe having a list of a few side effects, but reassurance about them, how they can be treated, like that kind of thing, instead of just saying all negative things about them. So with the drooling, like, with the clozapine, it would have been really helpful to read something and say, yeah, that might happen, but if you take Kwells it won’t happen at all. ***P26 Lived Experience***    But in terms of information, not hugely, no, it was more me asking really. However, what was good was there was no pressure to be on something, and I think it was a joint decision because I was completely in control of my decision. And I did discuss this with my husband as well, I think had it been something where I had it been something where I have to be on it for the sake of everybody and my health I would have had to reassess that. However, I was in a fortunate position where I was on the road to recovery and I did… Obviously, don’t get me wrong, I probably would have been better on it, but, to be honest, there wasn’t any major impact from not being on it. ***P27 Lived Experience***    And I think you’ve got to be careful because you don’t want to scare people half to death with the horror stories of antipsychotic medication, but you also want to be open and honest that these are serious meds and they’re not to be sniffed at. I think it’s that openness. I got some feedback from a carer about two months ago actually and she commented on how I reassured her son that we wouldn’t tolerate the potential side effects of the antipsychotic I was giving at the time, that we wouldn't expect them to put up with those side effects so please don’t… It’s sort of expectations, isn’t it? If you underplay and the client has a negative experience and you’ve not acknowledged that then, I just think you lose the trust right from the off. You’ve got to be upfront and got to be… Like I say, with that lad it was a case of look, you might not experience these, but if you do, I accept they’re horrendous, and we won’t let you tolerate that and we’ll think again and we’ll do something different. ***P31 ACP***    In all honestly probably very little (information) is what they get. Apart from a discharge summary of what they've been discharged on. And if they're very fortunate they'll hopefully get some information whilst they're an inpatient about what they're on and what it's for, potentially how it works. We have in general practice they creation of structured medication reviews, which is just a way of saying we can talk about your medicines in-depth, as well as look at the requirements, the expectations of those medicines from both a patient and a healthcare professional perspective. So we can go into a lot more detail. I think when we think holistically the question always starts with what do you know so far as a patient about your medicines? And do you want to know more? Because as a pharmacist, I've got so much information that I could give you. But the questions really are do you need that information or do you want that information? ***P33 Pharmacist***    I’ve had enough patients contact me to discuss their medications for me to doubt that the patients are involved in those decision-makings. So, I have enough patients contact me about their medication, I’ve had enough specifically ethnic minority patients who have been put on a drug but they don’t fully understand why, they just have to take it because they don’t have a choice in taking it. To know that I don’t think patients are fully being counselled or fully being given options or fully discussing to them why they are being put on a certain drug over another drug. That baffles me a little bit as a GP because with any patient that I am starting an antidepressant on or propanol on, I always have a discussion of going, I recommend sertraline because you have a mixed depression/ anxiety picture and this would probably be the better fit or mirtazapine because a big feature of you is sleep difficulties. Propanol because actually I think you are in a panic attack avoidance cycle and I’m hoping propanol will break that cycle and then we will review things. So, I explain to patients why they are taking something but I don’t feel that necessarily comes from my counterparts when they are an in-patient. ***P35 GP***    So, I suppose, for patients they will, as standard they get a discharge summary anyway. And then it just depends on the information on there. And it's where the patients can understand the content of the letters. Because it will have about their stay and changes to things but I think my impression is that patients often find it quite confusing. And it's, even bits like, I suppose as a healthcare professional I'm like, here's a list of medications, here's a bag of medication basically, and marrying the two up. But even that in itself, patients can find really tricky. And it's just knowing your patient as well. ***P37 Pharmacist*** | |
| C3. Patient and carer empowerment or disempowerment | Variable patient or carer involvement included patients or carers feeling not listened to and disempowered. |
| So it's about sometimes if that's not accurate, so say, for example, on some of these forms sometimes we get told patients are completely independent with their own medicines and we get there and patients aren’t I suppose, and then it's like, right, what do we do and how do we make this safe for the patient, and how do we have the best impact possible essentially. Because sometimes I'll go and review, sometimes, we have something called reablement which is a service provided by (City) City Council, it's almost like a short-term care, but the idea is to help someone back to full health. So within that if reablement are already involved but not involved with meds prompt, sometimes we can just say, can you help out with this and then they'll pick the patients up and things like that, which is good. And they're very useful to you, because the aim is to try and get patients back to whatever their previous level of independence was. So it's good to get patients back to that way, because it's better for all parties involved, for want of a better term. So sometimes I'll go and see patients and complete counselling and look at how they are managing their medicines and if there's things that we can optimise and improve for patients just to try and help them out a little bit. ***P37 Pharmacist***  Yeah, they probably could've. I mean I have a big upset, once he got sectioned, they refused to let me see him. And this was before the restrictions came in around COVID. It was 6th March he was sectioned and they just refused point blank. They said it would distress me. And I was there saying but hang on a second, how do you know what I can cope with? Have you spoke to my GP, or not? So yeah. But they were saying oh, well, it'll distress you to see him. And as it happens, I didn't see him right up till his death, because of what happened […] Although I find it more distressing not seeing him. ***P13 Carer***    And I think one of the other things that I do have a bit of an issue with is when a patient comes into hospital, we take complete ownership of their medicines. So they can’t self-medicate, they are the patients that understand most about how their diabetes is managed, when they might need their inhaler, why something might cause them indigestion if they have it before they have their breakfast. And actually, you take that empowerment away from them, and then all of a sudden, you’re given this bag of drugs, and say, okay, off you go now, off you go home, and you just carry on doing what you’re doing, and we’ve added an extra ten drugs as well, and we’ve removed some of the ones that you almost use as a prompt. So, I don’t think we help ourselves either, really. Yeah. ***P14 Pharmacist***  So, because it was kind of like a rehab ward, kind of thing, a couple of months before I was discharged, they started kind of letting me have a bit more control over taking my meds and remembering to take them. So initially it was kind of, instead of being prompted to go to the meds room and get the meds, it’s like remembering to go myself. And then they gave me my meds for the day, in like a single blister pack, and they’d do randomised checks during the day, each day, to check that I was on track with them, and that kind of thing. So then it went from giving me one day at a time, to I’d get a week, and they’d check me once a day with the meds, rather than a few times. So it was quite a graded process, which really helped actually. And now I still have my meds in a blister pack, which is really good. Because obviously, there’s so many, I would be putting them out myself all the time, but when they’re in a blister pack, they’re just ready for you to take and I don’t have to think about it. ***P26 Lived Experience***    I want to make an informed decision, and I want to be treated like I’m not stupid, you know? I want to be treated like, that I can tie my own shoe-laces, you know what I mean? Sometimes they really do treat you like you don’t have a brain, and it’s really patronising. So yeah, I want them to sit down there and give me an extra ten minutes of their time and tell me everything. And that might make me sound awful, but I think we deserve it as much as a lawyer or doctor, or anyone like that. ***P28 Lived Experience***    One thing that I really wanted to avoid was I didn't want to be seen as like a difficult patient. I didn't want to be so insistent on switching that then I became seen as, I don't know, someone who's…I don't know. I just get the impression that if you're seen as a demanding patient or as a patient who thinks they know best or something, that that can kind of impact how you're seen clinically I think. So I wanted to avoid that. I didn't want to end up being kind of perceived as someone who's hard to work with. ***P29 Lived Experience***  And I still struggle, like, on many days, you know, to manage because of the dosage of the medication. And I have brought this across many times, you know, with my team, my CPN. I've told them many times but I think the problem with all these experiences that…they do not want to listen to me. Like, I try, I try, I try, I explain my situation, even with my mental health advocate. But they don't really pay any attention to what I'm explaining to them or they're not listening to me*.* ***P30 Lived Experience***    Patients are all different, some people will definitely go and search for those answers themselves. Some won't. And I think because I always try and get in touch anyway, I think patients are always grateful. Sometimes it's a five-to-ten-minute conversation and they're like, great, I'm so happy and, sometimes it will be a bit more than that […] patients are surprised to hear from me sometimes because they're like, no one's ever spoken to me about my medicines and provided this information. And I think that’s always the thing, it's always a surprise, but it's always a good surprise because it's like, yes, I do want to talk about this. Because I think medication is so important to people for lots of different reasons and it can affect their lives in lots of different ways. ***P37 Pharmacist***    And the (place) based were absolutely useless. I was getting really, really bad, I wasn't sleeping, I was seeing things, I was hearing things. I was terrified. I was very ill. And they kept promising me that I would have a psychiatry appointment to get help with this, to try to get some kind of medication, some kind of help, and they literally…they told me they'd get me one and four times then they said they weren't able to. They told me it was going to happen and then withdrew it. Meanwhile I'm spiralling, I was having issues with drinking and coke. I was begging for help. Not to be…I don't mean this to be triggering, but I ended up cutting myself in front of them, because after the last time they told me that they couldn't help me, and all they could do was show up to my house for 20 minutes, and the only thing left that I could do was go to A&E. And if you have a diagnosis of BPD at A&E they don't tend to really do very much. So I had nothing. ***P5 Lived Experience***    No. I never felt like I had any real clear pathway that was going to be followed. I was very confused the whole time. I felt lied to constantly because even if they weren't intending to deceive me, I was just constantly told one thing and then something else would happen. It was…there was never a clear expectation. There was never a clear pathway laid out to me. I was told every day for two and a half weeks that the psychiatry team would come to see me that day, and it wasn't for two and a half weeks that they did. I was self-harming, I had cuts everywhere and the nurses weren't doing anything. ***P5 Lived Experience***    They'd asked me to give ideas, like they'd say, oh, what helps you cope and the few things I'd listed they would write down and be like, oh, this is your safety plan. But there was never any consideration and I always felt like I had to come up with the ideas myself, and it's like I'm actually coming to you asking you to help me find coping mechanisms. If I could find them myself, I wouldn't be here. […] I'm a fairly introspective, self-reflecting person, if I can't do something I can't do something, I will say it straight out. […] I begged, I cried, I fucking cut myself trying to get them to listen. ***P5 Lived Experience*** | |
