# Supplementary material for: Exploring Medicines Optimisation and Safety in the Community Following Mental Health Hospital Discharge: A Qualitative Interview Study
Source: Health Expect. 2025 Dec 25;29(1):e70535. doi: 10.1111/hex.70535 (PMC12741015; doi:10.1111/hex.70535)
Supplement: Supplementary file 3 — Supplementary_File_S3_Reporting_Framework_on_lived_experience_engagement. [file HEX-29-e70535-s003.docx]

**Exploring Medicines Optimisation and Safety in the Community Following Mental Health Hospital Discharge: A Qualitative Interview Study**

**Supplementary File - S3 - Reporting Framework on lived experience engagement (Sheikhan, 2024)**

| **Reporting Framework on lived experience engagement** | | | **Response** |
| --- | --- | --- | --- |
| Areas to report  and reflect on | Guiding questions | Additional reflections |  |
| Who was involved? | Who were the PWLE/F engaged? Are there any descriptive factors that are relevant to the research question? | For PWLE/F partners, consider reflecting on how you want to be referenced in papers | The research team included a person with lived experience (FN) who is a co-author of the study. FN had lived experience of being discharged from inpatient mental health care and managing medications in the community.  In reflecting on how to be referenced in the papers FN added *“My actual title is Reverend but in discussion with the other researchers I choose not to use this because it may have detracted attention away from the research. The other researchers also did not use their titles”*  In addition our study advisory group included a person with lived experience and a carer. |
| What were the activities, roles, and responsibilities? | What were the specific activities for engagement? What were the roles and responsibilities of the PWLE/F partners? What was the level or extent of engagement throughout the project? Was there any training provided for the PWLE/F engaged? | Consider reflecting on your social positioning and the  role you have on the research team, in particular, the roles  of co-authors. | FN acted as a full member of the research team. They were involved in developing the research, including the ethics application. They gave feedback on study documents including participating information sheets, recruitment adverts and topic guides for interviews. They were engaged in analysis, reading transcripts and making contributions to analysis meetings. They also commented on drafts of the manuscript as it was developed. As such they are a co-author of the manuscript. No training was given partly because of resources and partly because FN had extensive previous research experience. FN contribution was to the study was interrupted by further illness and reflected *“I found the researchers understanding when I suffered a relapse during the research study”.*  The study advisory group members met twice during the lifetime of the project along with key members of the research team and gave feedback on the progress of the research and supported the research team to resolve any challenges. |
| How did you go about the engagement process? | What did the engagement process look like?  What were the decision-making processes and feedback loops? How were relationships built?  Was there a terms of reference? What was the engagement context (e.g., virtual, in person)? Did you compensate your PWLE/F partners? How did you recruit PWLE/F partners? Were there any accommodations provided to make the space more inclusive? Are there any influencing factors that facilitated the engagement process? Any factors that acted as barriers?  What were the solutions and lessons learned? How were power imbalances addressed? | Consider reflecting on why you are engaging PWLE/F in the first place, how power appeared during the engagement process, and any efforts the team made to minimize the power dynamic between researchers and PWLE/F partners. | The research team considered that having a PWLE was vital to the research to provide understanding of the specific challenges related to discharge from mental health inpatient care and medicines. At first we recruited a lived experience person who was involved in early stages of development of the study. They could no longer continue in the role so FN was then recruited via the research team’s professional network within the same institution. FN attended online research team meetings and in person data analysis sessions as an equal contributor. FN reflected *“ I recognised myself as an equal part of an academic team which had acknowledged the potential for power dynamics but made effort to eliminate those”.*  FN and the study advisory group members were compensated for their time spent at meetings and for required preparatory activities, including the PWLE time spent reading and analysing interview transcripts. |
| When did engagement occur? | What did the timeline for engagement look like? At what stage(s) of the research process were PWLE/F engaged? What was the frequency of meetings? | Consider reflecting on the timeline for engagement early on and if sufficient time was set for engagement activities. | Research team meetings occurred monthly to which the FN was always invited and were flexibly online or face-to-face. Other engagement was when required as data analysis progressed.  The study advisory group members attended two one-hour online meetings during the lifetime of the project to constructively review progress and support the project. These meetings were at key project milestones: participant recruitment and data analysis/interpretation. |
| What was the impact? | What was the impact of engagement on the research project (e.g., recruitment, analysis)? What was the impact on PWLE/F? What was the impact on researchers? Was engagement evaluated? What were the experiences of PWLE/F? | Consider reflecting on the value engagement has to the PWLE/F engaged. Was the experience good for PWLE/F? How did it benefit them? Were there any negative impacts? | The impact of having FN as a PWLE was essential in providing a different perspective to the planning and conduct of the study. In particular during data analysis this input helped in providing reassurance to the research team that data collection and analysis were proceeding in a way that sensitively captured and represented the lived experience perspective.  FN further reflected: *“As part of this research I gained insight into the academic process. My experience was a very positive one. I was pleased that academic knowledge was shared . I never felt any pressure to frame my experience in academic language and my contributions to the project were actively encouraged and treated with care. Additionally being part of this research was a protective factor in my recovery from an acute episode of ill health”*  The impact of the study advisory group was to reassure the research team that the project was on track and appropriate in its approach. Additionally the study advisory group helped to troubleshoot recruitment challenges by supporting the team to identify new avenues*.* |
